# WGG: a graphical tool for visualizing genome structures using SNPs detected by next-generation sequencing

**DOI:** 10.1270/jsbbs.25080

**Published:** 2026-06-11

**Authors:** Marina Takata, Miki Sakemoto, Mutsuki Oishi, Makishi Hara, Masahide Yoshizumi, Tenta Segawa, Rintaro Harada, Hiroki Takagi

**Affiliations:** 1 Ishikawa Prefectural University, 1-308 Suematsu, Nonoichi, Ishikawa 921-8836, Japan; 2 Ishikawa Agriculture Research Center, 295-1 Saida Bo, Kanazawa, Ishikawa 920-3198, Japan

**Keywords:** next-generation sequencing, genome structure, whole genome selection, genotyping, rice, *Brassica rapa*

## Abstract

Monitoring genome structure provides a great advantage for efficiently and accurately advancing breeding. However, genotyping using DNA markers distributed across the genome is time-consuming and labor-intensive for breeders. Here, we developed a graphical user interface application, “WGG: Whole Genome Genotyping”, enabling rapid and easy visualization of genome structures based on single nucleotide polymorphisms (SNPs) detected by resequencing with next-generation sequencing short reads. The accuracy of WGG is enhanced by selecting SNPs from parental resequencing data and determining the majority genotype within each genomic region. As proof of principle, we efficiently developed near-isogenic lines in rice by monitoring genome backgrounds with WGG in intermediate generations. The identification of residual donor regions with WGG facilitated their elimination using DNA markers designed at these loci. Furthermore, we confirmed the applicability of WGG in rice recombinant inbred lines and heterozygous plants, *Brassica rapa*. In addition, WGG was confirmed to run smoothly on a standard desktop PC even with sufficient sequencing data, making it practical for breeders. WGG will be a powerful tool for visualizing genome structures in breeding intermediate generations, enabling whole-genome selection that will accelerate breeding in diverse crops.

## Introduction

The global population is projected to reach approximately 10 billion by 2050 (United Nations Department of Economic and Social Affairs 2019), while climate change continues to accelerate environmental fluctuations worldwide (IPCC 2023). To secure a stable food supply and improve crop adaptability under such changing conditions, accelerating crop breeding has become an urgent challenge for modern agriculture.

Among the various breeding approaches, marker-assisted selection (MAS) has proven to be one of the most effective and reliable strategies (Collard and Mackill 2008, Collard *et al.* 2005, Hasan *et al.* 2021). MAS enables the selection of individuals carrying desirable alleles through the use of DNA markers tightly linked to genes controlling target traits. This genotypic selection approach reduces the dependence on time-consuming phenotypic evaluations and enables selection at early developmental stages, thereby shortening the breeding cycle while lowering production costs and land requirements. Consequently, MAS has been extensively applied across a wide range of crops to improve not only qualitative traits but also key quantitative traits, such as disease resistance, tolerance to abiotic stresses, and yield-related characteristics (Cobb *et al.* 2019, Fujino *et al.* 2019, Laxmi *et al.* 2022).

In modern breeding, the development of near-isogenic lines (NILs) has been widely adopted as a practical strategy that utilizes the advantages of MAS. This approach enables the rapid and precise introgression of genomic regions controlling desirable traits into the genetic background of elite cultivars. NILs developed in elite cultivars with well-established cultivation systems retain the superior agronomic background and can therefore be readily adopted by farmers (Fukuoka *et al.* 2009, Ishizaki *et al.* 2005, Matsumoto *et al.* 2022). Moreover, in rice breeding, such NILs developed in a common elite background have been further utilized for gene pyramiding, in which lines are crossed to combine multiple beneficial genes (Ashikari and Matsuoka 2006, Fukuoka *et al.* 2015, Ookawa *et al.* 2022). Taken together, the development of NILs using MAS and their subsequent use in gene pyramiding constitute a highly efficient approach in crop breeding.

However, the development of NILs is often challenging because it requires repeated backcrossing between a donor line carrying the target gene and an elite recurrent parent, in order to replace the donor genetic background with that of the elite cultivar. The number of crosses with an elite cultivar is generally determined based on the theoretical probability of recovering the recurrent parent genome. In diploid species, the proportion of the recurrent parent genome increases according to the formula 1 – (1/2)^n^ per cross, where n is the number of crosses. Consequently, at least five crosses with the recurrent parent are theoretically required to achieve a substitution ratio of 95% or higher. While such a number of crosses can be accomplished in species that are easy to hybridize and allow multiple generations per year, it is difficult to implement in crops with complex crossing systems or long generation times (Degen and Müller 2023, Kitamoto *et al.* 2023). Therefore, reducing the number of crosses required for NIL development remains a major challenge in crop breeding.

One practical solution to this challenge is to monitor the genetic background at intermediate generations during NIL development. Monitoring the genetic background allows breeders to identify individuals whose genomes already contain a high proportion of the recurrent elite parent genome. By precisely selecting these individuals, breeders can preferentially advance lines that are closer to the elite genetic background than expected from theoretical probabilities alone. This targeted selection reduces the number of crosses required to achieve a sufficient level of genome substitution. However, developing genome-wide DNA markers or arrays for each crossing combination is costly, and genotyping remains labor-intensive (Bassil *et al.* 2015, Clarke *et al.* 2016, Gupta *et al.* 2008, McCouch *et al.* 2002).

Recent advances in next-generation sequencing (NGS) technologies have dramatically reduced the time and cost required to detect genome-wide DNA polymorphisms with high accuracy (Deschamps and Campbell 2010, Goodwin *et al.* 2016, Mardis 2017). In particular, single nucleotide polymorphisms (SNPs) can now be accurately and efficiently detected from resequencing data generated by low-cost short-read sequencing (Kumar *et al.* 2016). Therefore, genome-wide SNP information obtained through NGS can be effectively applied to assess the genetic background of individuals at intermediate stages of NIL development with high precision and at a practical cost. This information enables breeders to select individuals possessing a high proportion of the recurrent parent genome.

Several bioinformatics tools for analyzing resequencing data, such as VIVA (Tollefson *et al.* 2019), VCFshiny (Chen *et al.* 2023), and GenoSee (Hashimoto 2024), have been developed for visualizing genotypes at SNP positions. These tools mainly require variant call format (VCF) files as input (Danecek *et al.* 2011). VCF files are generated from BAM files, which are binary alignment files that are produced as the primary output files during the resequencing process (Danecek *et al.* 2021). Therefore, preparing VCF files requires an additional processing of BAM files, which is time-consuming and labor-intensive, although VCF files are lighter and easier to handle once generated. In addition, few software tools have been developed to efficiently visualize genome-wide background composition from resequencing data rather than focusing only on local genotypes. Hence, there is still a need to develop a tool that is practical for breeders who are directly engaged in NIL development.

In this study, to accelerate gene pyramiding by MAS, we developed a whole-genome genotyping (WGG) application. WGG performs genotyping at genome-wide SNP positions and visualizes the genome structure in progenies derived from crosses. We demonstrated its applicability not only in rice NIL development but also in recombinant inbred lines (RILs) and in the heterozygous crop *Brassica rapa*. Finally, we validated the effectiveness of WGG-assisted MAS in gene pyramiding by developing a pyramided line and evaluating its agronomic traits.

## Materials and Methods

### Implementation environment of WGG

WGG was implemented as a Python-based graphical application. The program was built into a standalone executable using PyInstaller, allowing it to run without requiring a separate Python environment. WGG requires samtools (v1.18 or later) and bcftools (v1.18 or later) for BAM and VCF file processing (Danecek *et al.* 2021, Li *et al.* 2009). The parameters used in WGG were as follows: minimum read depth of 6, window size of 2 Mb, step size of 100 kb, and a minimum of 3 SNPs per window. In addition to the macOS version of the WGG application, a Windows build incorporating a Linux runtime environment and a command-line interface (CLI) version for Linux PCs are also available from the same GitHub repository: https://github.com/takatamarinaalt/wgg-gui/releases/tag/v1.0.1.

### Developing BAM files for WGG analysis

For rice, BAM files were generated by aligning short reads to the IRGSP-1.0 reference genome (International Rice Genome Sequencing Project 2005) using bwa-mem2 v2.2.1 (Vasimuddin *et al.* 2019). For *B*. *rapa*, BAM files were generated using the Chiifu v4.1 reference genome (http://brassicadb.cn/#/), applying the same alignment settings as used for rice.

### Computational environment

All analyses were conducted on a Mac mini with an Apple M2 8-core CPU and 8 GB RAM running macOS Sonoma 14.2.

### Obtaining whole-genome sequence data

NGS short reads used in this study were obtained either from our own sequencing or from public databases. For in-house sequencing, libraries were prepared using the NEBNext Ultra II DNA Library Prep Kit for Illumina (New England Biolabs, USA) and sequenced on an Illumina Hiseq 6000 or NovaSeq X platform to generate 150 bp paired-end reads. In addition, publicly available resequencing data were downloaded from the NCBI Sequence Read Archive (SRA; Leinonen *et al.* 2010). All accession numbers of the datasets used in this study are provided in Supplemental Table 1.

### Genetic analysis in rice

Genetic analysis by QTL-seq in rice was performed using progeny derived from a cross between two cultivars showing different phenotype. QTL-seq analysis was conducted following the protocol described by Segawa *et al.* (2021).

### Field evaluation of the phenotype in rice

Field evaluation of rice was conducted in the paddy field of Ishikawa Prefectural University (IPU), Ishikawa, Japan, in 2025. Plants were grown under standard paddy field conditions with a basal fertilizer application of 8 kg nitrogen per 10 a. Seeds were sown on March 28, and seedlings were transplanted on April 22. Agronomic traits, including heading date, culm length, panicle length, number of spikelets per panicle, 1,000-grain weight, percentage of ripened grains, and grain yield, were evaluated.

## Results

### Principle of WGG

The principle of whole-genome genotyping (WGG) is summarized as follows (Fig. 1). The WGG application requires two types of input data: BAM files from P1, P2, and the sample, and the FASTA file used to generate those BAM files.

First (Step 1), to reduce memory usage, WGG divides the input BAM files into smaller segments, which are then processed sequentially. Next (Step 2), homozygous SNP positions between the parental lines are identified from resequencing data. Then (Step 3), sample genotypes are determined based on SNP-index values at these identified positions. When the SNP-index value is 0 or 1 and coverage is sufficient, the genotype is classified as homozygous and designated as either P1- or P2-homozygous, depending on the allele. When the SNP-index value falls between 0 and 1 and follows the expected distribution from F_1_ simulation data, the position is classified as heterozygous. In our F_1_ simulation data, a minimum coverage of six reads was required to distinguish the SNP-index pattern between homozygous and heterozygous sites. Subsequently (Step 4), a sliding window analysis is performed by calculating the frequency of each genotype within a given genomic interval (window size). The predominant genotype in each window is assigned as the representative genotype for that region. Finally (Step 5), the overall genotype composition across all chromosomes is visualized as a color-coded chromosomal display. Contiguous regions with the same genotype are filled with the same color, whereas the boundaries between different genotypes are displayed with color gradients.

The WGG application has been developed as a graphical user interface (GUI) program and is freely accessible on GitHub (see Materials and Methods). Through the GUI, users can specify input files and execute analyses via simple point-and-click operations, without requiring command-line input (Fig. 2). In addition, parameters such as the analysis depth threshold, window size, and the minimum number of effective SNPs within a window can be freely adjusted by the user.

The output data generated by the WGG application include the following: graphical files showing the genome structure, genotype calls for all SNP positions used for the sliding-window analysis, the proportion of each genotype, and the SNP density in each genomic region. In addition, WGG generates text files that include the genotype data from the sliding window analysis used to construct the genome structure graph, as well as the genotype information of the sample at all homozygous SNP positions between P1 and P2.

### Applying whole-genome genotyping in practical rice breeding

To demonstrate the utility of the WGG application, we applied it to an introgression breeding program aimed at improving heading date and conferring blast resistance in the sake-brewing rice cultivar ‘Ishikawa Sake 68’ (IS68). ‘IS68’ was developed and is cultivated in Ishikawa Prefecture; however, its late flowering habit restricts cultivation in the northern Noto region. In addition, its low resistance to blast disease requires considerable labor for disease prevention.

For the improvement of heading date, we targeted the *Hd16* and *Hd17* alleles derived from ‘Ishikawa 65’ (I65). These alleles had been identified through genetic analysis of progeny from a cross between ‘IS68’ and ‘I65’ (Supplemental Fig. 1). For blast resistance, we introduced the resistance gene *pi21*, as reported by Fukuoka *et al.* (2009), from ‘Ishikawa 73’ (I73). The overall breeding scheme is illustrated in Fig. 3.

First, ‘IS68-E’, carrying the ‘I65’-derived *Hd16* and *Hd17* alleles in the homozygous state within the ‘IS68’ genetic background, was developed through five successive crosses with ‘IS68’. The presence of the ‘I65’-derived *Hd16* and *Hd17* alleles was confirmed at each generation using gene-specific DNA markers (Supplemental Table 2). Similarly, ‘IS68-R’ was developed to introduce the blast resistance gene *pi21* from ‘I73’ through six successive crosses with ‘IS68’. In ‘IS68’-R, the *pi21* allele from ‘I73’ was confirmed to be in the heterozygous state using DNA markers.

‘IS68-E’ and ‘IS68-R’ were then subjected to WGG to confirm the genotypes at the target gene regions and to evaluate the proportion of the ‘IS68’ background genome (Fig. 4a, 4b). The analysis confirmed the expected genotypes and revealed that they retained 95.4% and 96.4% of the ‘IS68’ background genome, respectively (Supplemental Fig. 2). Additionally, residual donor segments were identified: ‘IS68-E’ carried ‘I65’-derived segments on chromosomes 3, 5, 7, and 9, whereas ‘IS68-R’ retained ‘I73’-derived segments on chromosomes 8 and 11. When the analysis parameters of the sliding-window analysis in WGG, such as window size and the minimum number of SNPs per window, were modified, the overall genome composition and genotype patterns remained consistent (Supplemental Fig. 3a, 3b). However, when the sliding-window size was too small, the insufficient number of SNPs per window allowed irregularly detected SNPs to affect the inferred genome composition. (Supplemental Fig. 3c, 3d). Regarding the residual donor segments identified above, those in ‘IS68-E’ and ‘IS68-R’ were located at different loci, suggesting that these segments could be eliminated through progeny derived from a cross between ‘IS68-E’ and ‘IS68-R’.

Among the 10 F_1_ individuals obtained from a cross between ‘IS68-E’ and ‘IS68-R’, five carrying all three target genes in the heterozygous state were selected. Subsequently, one individual was further selected after confirming the absence of the residual donor segments on chromosomes 8 and 11 derived from ‘I73’, in which ‘IS68-R’ had been heterozygous. From this selected F_1_, 129 F_2_ individuals were developed. Among the F_2_ population, one individual carrying homozygous genotypes at all three target genes, as confirmed by DNA markers, was selected and designated as ‘IS68-ER F2’. WGG analysis of ‘IS68-ER F2’ revealed that two large residual donor segments, each exceeding 1 Mb, derived from ‘I65’ (chromosome 5: 21.5–22.6 Mb and chromosome 7: 25.7–29.6 Mb), were still retained in the heterozygous state (Fig. 4c). To eliminate these segments, MAS was performed in the selfed F_3_ generation using DNA markers within the donor regions. An individual carrying the homozygous ‘IS68’ genotype at these loci was successfully obtained and designated ‘IS68-ER’ as the final breeding line.

### Application of WGG in earlier generation for rapid introgression breeding

We employed a strategy similar to that used for ‘IS68’ to improve heading date and blast resistance in the cultivar ‘Yumemizuho’, an early-maturing staple rice cultivar from Ishikawa Prefecture. First, heading date related genes were identified through genetic analysis of ‘Yumemizuho’ and ‘Ishikawa 67’ (I67) (Supplemental Fig. 4). This analysis indicated that the ‘I67’ alleles at *Hd18* and *OsMADS50* confer early heading in ‘Yumemizuho’. Based on these findings, we initiated a breeding program to introgress the ‘I67’-derived *Hd18* and *OsMADS50* alleles for heading date together with *pi21* from ‘I73’ into the ‘Yumemizuho’ genetic background (Supplemental Fig. 5).

In this breeding program, WGG was applied at earlier generations than in the ‘IS68’ gene pyramiding scheme to accelerate introgression breeding. ‘BC_2_F_2_-E’ and ‘BC_3_F_2_-R’ were developed using ‘Yumemizuho’ as the recurrent parent and were then subjected to WGG analysis (Fig. 5a, 5b). As expected, due to the fewer backcrosses with ‘Yumemizuho’, ‘BC_2_F_2_-E’ retained larger residual donor segments compared with ‘BC_3_F_2_-R’. However, similar to the case of ‘IS68-ER’, these residual donor segments did not overlap between ‘BC_2_F_2_-E’ and ‘BC_3_F_2_-R’, suggesting that they could be eliminated in progeny derived from a cross between ‘BC_2_F_2_-E’ and ‘BC_3_F_2_-R’.

To pyramid the three target genes while eliminating residual donor segments, approximately 160 F_2_ individuals were developed from an F_1_ plant carrying all three target genes in the heterozygous state. Among them, two F_2_ individuals (‘F_2_-85’ and ‘F_2_-83’) carrying all three target alleles in the homozygous state were selected using DNA markers. Subsequently, both F_2_ plants were selfed to develop ‘F_3_-85’ and ‘F_3_-83’ lines, which were subjected to WGG analysis (Fig. 5c, Supplemental Fig. 6). WGG analysis revealed that the ‘F_3_-85’ line recovered a larger portion (79.9%) of the ‘Yumemizuho’ genome than ‘F_3_-83’ (72.5%) and was completely homozygous across the entire genome. Therefore, the progeny derived from the ‘F_3_-85’ individual analyzed by WGG was defined as the final breeding line, designated ‘Yumemizuho-ER’ (Yume-ER).

To confirm the effects of gene pyramiding, we evaluated the heading date and blast resistance of ‘Yume-ER’ (Fig. 5e–5g). The heading date of ‘Yume-ER’ was significantly earlier than that of ‘Yumemizuho’ and almost identical to that of ‘I67’. In addition, artificial spray inoculation confirmed that ‘Yume-ER’ exhibited strong blast resistance similar to the donor parent ‘I73’, whereas ‘Yumemizuho’ showed severe symptoms. Furthermore, other agronomic traits evaluated under field cultivation indicated that ‘Yume-ER’ exhibited similar characteristics to ‘Yumemizuho’, except for seed weight (Supplemental Fig. 7). Consequently, the phenotypic effects of the introgressed alleles in ‘Yume-ER’ were clearly confirmed.

### Broader applicability of WGG

We confirmed the applicability of WGG in recombinant inbred lines (RILs) for determining accurate genome structures. As an example, we applied WGG to the published sequence data of six rice RILs derived from a cross between the japonica cultivar ‘Hitomebore’ and the indica accession ‘WRC17’ reported by Shimizu *et al.* (2022) (Fig. 6a, Supplemental Fig. 8). The WGG analysis revealed that all RILs exhibited mosaic genome structures composed of parental segments. Importantly, all RILs correctly retained the locus containing the susceptible *Pias* and *Pita* allele derived from the parent ‘Hitomebore’, consistent with the genotype data reported in that study. In addition, some RILs showed unexpected heterozygous regions even though they had been advanced to the F_5_ generation or beyond, indicating the high sensitivity of WGG for detecting residual heterozygosity. These results demonstrate that WGG accurately identifies parental genome segments and reconstructs genome composition in homozygous inbred lines.

Additionally, we confirmed the applicability of WGG in heterozygous individuals. As an example, we applied it to an NIL, ‘NIL-496’, of the *B. rapa* turnip cultivar using the published sequence data reported by our group (Segawa *et al.* 2021) (Fig. 6b). ‘NIL-496’ carries two ‘Akamaru’ (AKA)-derived genomic regions controlling turnip skin color in the genomic background of ‘Kanazawa Ao Kabu’ (KAN). The WGG analysis successfully visualized the expected genome composition, showing two ‘AKA’-derived segments within an 85.4% ‘KAN’ background, although minor noisy misgenotyped regions were observed.

Taken together, these results confirm that WGG is effective for analyzing genome structures even in heterozygous diploid plants.

### Evaluation of WGG performance on macOS under different data sizes

To evaluate the performance of WGG in a standard desktop environment (Mac mini, Apple; 8 GB (gigabyte) RAM), we measured the processing time and memory usage (RSS) for the samples described in Fig. 6a as a test case (Fig. 7). When the same dataset used in Fig. 6a (a total of 28 GB of BAM files generated from 40.6 Gb (gigabases) of resequencing reads) was analyzed using WGG, the total processing time was <41 min, and the maximum memory usage was 1.32 GB during genome structure visualization. When BAM files of different sizes, generated by reducing the amount of sequencing reads to approximately 12×, 6×, and 3× genome coverage in both parents and the sample, were applied to WGG, the total processing time decreased in proportion to the read depth (Supplemental Fig. 9). However, the genome composition analyzed at 3× coverage showed considerable noise, indicating that a sequencing depth of at least 6× is recommended for reliable results. The maximum memory usage was approximately 1.3 GB and remained almost constant regardless of the amount of sequencing data. These results suggest that WGG can be implemented efficiently on a standard desktop computer.

## Discussion

In this study, we successfully established an NGS-based WGG system. The WGG application enabled the visualization of precise genome structures by comparing parental alleles and determining the majority genotype within each genomic interval using a window-based analysis. WGG was effectively applied to whole-genome selection in a gene-pyramiding breeding program through the development of NILs. Furthermore, we confirmed its applicability across various diploid species and its usability on a standard desktop PC, suggesting that WGG can serve as a practical tool for breeders.

Previously published tools such as VCFshiny (Chen *et al.* 2023) and GenoSee (Hashimoto 2024) simply visualized sample genotypes based on Ref/Alt alleles from VCF files generated solely from the resequencing data of the sample. As a result, it was difficult to determine whether the Ref or Alt allele corresponded to P1 or P2, making it unclear whether the sample genotype at each locus was derived from the P1 or P2 parent. In contrast, WGG determines sample genotypes based on allele patterns detected in the resequencing data of P1 and P2, enabling accurate inference of the parental origin of each genomic region in the sample. Furthermore, when analyzing samples from highly heterozygous species, it is necessary to improve genotyping accuracy by carefully selecting analyzed SNP positions. In this study, WGG successfully revealed the expected genome structure in the heterozygous *B*. *rapa* sample (Fig. 6). However, noisy recombination signals can occasionally be detected in heterozygous plants due to SNP differences between the actual parental individuals used for crossing and those used for resequencing (Itoh *et al.* 2019). To address this issue, the WGG application includes a filtering option that utilizes sequencing data from the actual F_1_ individual obtained from the cross to filter out such discordant SNPs. This F_1_-based filtering function is particularly effective when the exact resequencing data of the parental individuals used in the cross are unavailable.

Additionally, the sliding-window analysis, which calculates the average genotype within a defined interval, helps reduce noise from local misgenotyping. In actual resequencing data, erroneous genotype calls often arise due to sequencing or alignment errors (Supplemental Fig. 3c), and if all such calls were accepted, the resulting genome composition would include numerous biologically unrealistic recombination events. In this study, genotyping was performed only for genomic regions that contained at least three SNPs. This threshold ensured reliable genotype estimation and resulted in clear visualization of genome structures (Supplemental Fig. 3a). The window size should be determined based on the SNP-density profile (Supplemental Fig. 2a) to ensure that each window contains a sufficient number of SNPs for reliable genotyping. This consideration is particularly important when using closely related parents, which results in low SNP density.

Another notable feature of WGG is ability to directly accept BAM files as input and operate through a GUI on a standard desktop PC. To reduce memory usage on such environments, the program automatically divides BAM files into smaller segments during processing. However, the current WGG system has limitations when applied to species with large genome sizes such as wheat (~17 Gb), chili pepper (~3 Gb), and onion (~16 Gb) (Finkers *et al.* 2021, Keeble-Gagnère *et al.* 2018, Kim *et al.* 2014). Although WGG was successfully applied to rice (~400 Mb) and *B. rapa* (~500 Mb) in this study, further optimization will be necessary for handling large-genome species on standard desktop environments by implementing fine-grained BAM file segmentation and improving memory management algorithms. WGG use BAM files as input because they can be easily generated using common platforms such as Galaxy without any command-line operations (Giardine *et al.* 2005, The Galaxy Community 2022). We verified that the results obtained using BAM files generated with the Galaxy platform were completely identical to those obtained using BAM files produced by BWA-MEM2 on the command line (Supplemental Fig. 10). In addition, the recently published DNA marker design program, DNAMarkMaker, also adopts BAM files as input (Segawa *et al.* 2024). Therefore, seamless integration between WGG and DNAMarkMaker enables breeders without bioinformatics expertise to efficiently perform MAS and accelerate the rapid development of NILs. Once DNA markers are designed at the residual heterozygous regions detected by WGG, these regions can be immediately eliminated through MAS in the backcross or subsequent selfing generations. Based on theoretical estimation, approximately 87.5% recovery of the recurrent parent genome can be achieved by the BC_2_F_2_ generation. During backcrossing, heterozygous regions are replaced by the homozygous genotype of the recurrent parent with a probability of 0.5. Therefore, if WGG detects residual heterozygous genomic regions in BC_1_F_1_ and DNA markers are designed at these regions, individuals retaining only the target donor genomic region can be selected with realistic probability in the BC_2_F_1_ generation. Even if some residual regions remain in BC_2_F_1_, they can still be eliminated in the BC_2_F_2_ generation. Consequently, the development of NILs can be accomplished by the BC_2_F_2_ generation, suggesting that subsequent gene pyramiding could also be completed within a remarkably short period. In rice, techniques enabling the advancement of up to four generations per year have been established by combining controlled flowering environments with embryo rescue (Tanaka *et al.* 2016). Therefore, it is realistic to expect that breeding could be completed within a single year by integrating WGG with MAS.

By applying WGG in combination with MAS, this study successfully developed two lines, ‘IS68-ER’ and ‘Yume-ER’. In both cases, flowering habit was modified and blast resistance was improved through gene pyramiding of three target genes. At present, many excellent cultivars have been developed and regionally branded in each prefecture in Japan, and ‘IS68’ and ‘Yumemizuho’ are among them. To maintain the value of these branded cultivars while further expanding their adaptability and utilization in accordance with variations in consumer preferences and environmental changes, the development of NILs and subsequent gene pyramiding will be essential breeding strategies. Taken together, whole-genome selection integrating WGG and MAS will be an indispensable approach for efficient NIL development and gene pyramiding in future breeding programs.

## Author Contribution Statement

MT developed the WGG application, performed genetic analysis in rice, and applied WGG to rice. MS performed genetic analysis in rice. MO, MH, and MY prepared the NGS libraries. TS evaluated the performance of WGG on a desktop PC. RH developed rice progenies. HT designed and supervised the study and wrote the manuscript. All authors played a role in writing the manuscript.

## Supplementary Material

Supplemental Figures

Supplemental Tables

## Figures and Tables

**Fig. 1.**
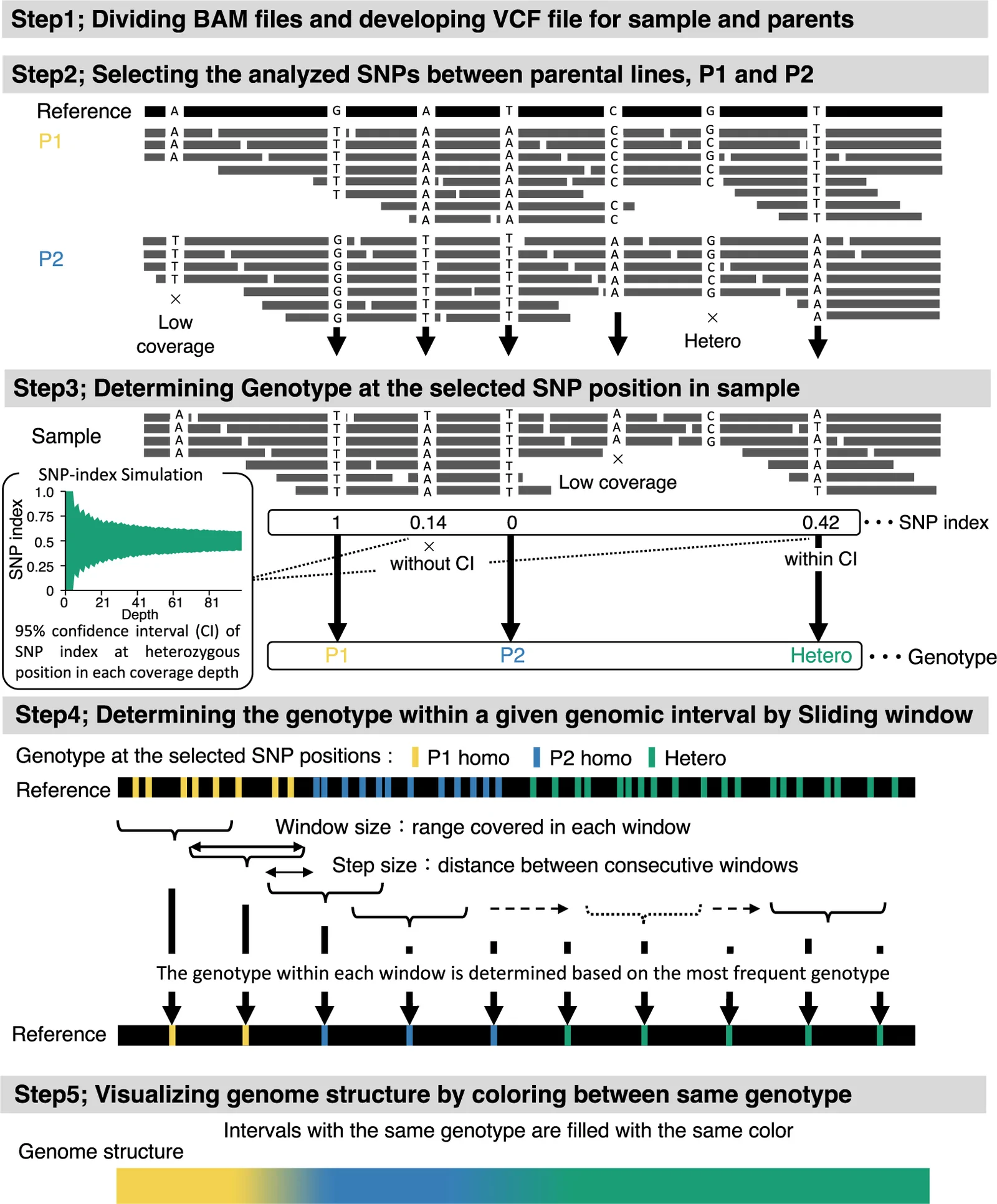
Workflow of the whole-genome genotyping (WGG) analysis. Step 1: BAM files are divided by chromosome, and VCF files for each chromosome are generated, including variants detected in samples and parental lines relative to the reference genome. Step 2: Homozygous SNP positions between the two parental lines (P1 and P2) are identified, and alleles at these SNPs are defined. Step 3: Genotypes of each sample are determined at the selected SNP positions identified in Step 2 based on the SNP-index values. Positions showing SNP-index values within the expected range of simulated F_1_ SNP-index values are classified as heterozygous, whereas positions with SNP-index values of 0 or 1 are classified as homozygous for P1 or P2. Step 4: A sliding-window analysis is performed to calculate the frequency of each genotype within a given genomic interval defined by user-specified window and step sizes. Step 5: The overall genome structure is visualized as a color-coded chromosomal map, where continuous regions of the same genotype are filled with the same color.

**Fig. 2.**
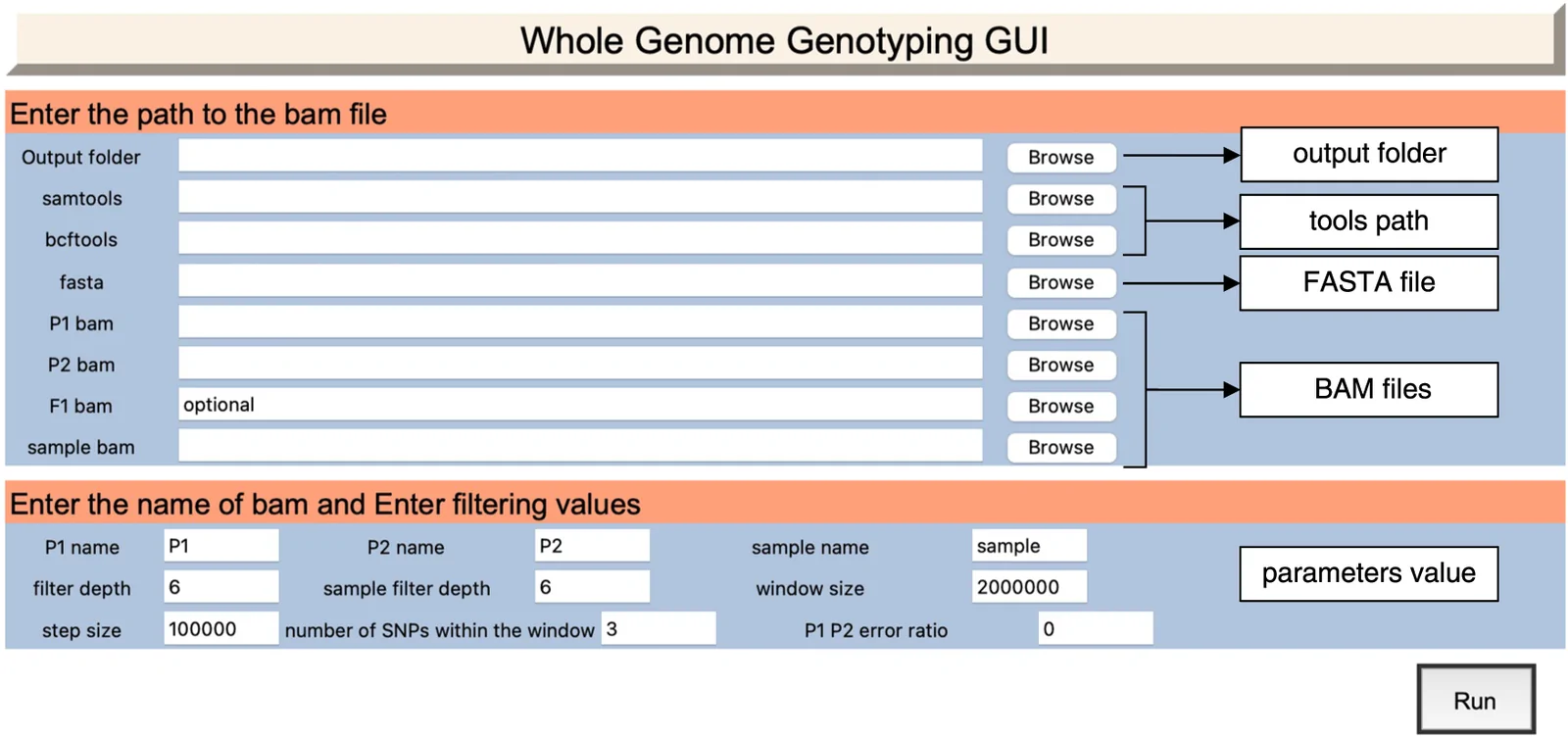
Input interface of the WGG application. In this interface, users can select the output folder, specify tool paths, and choose the FASTA and BAM files using the “Browse” buttons. Parameter values can also be freely modified by the user. The default settings correspond to those used in this study.

**Fig. 3.**
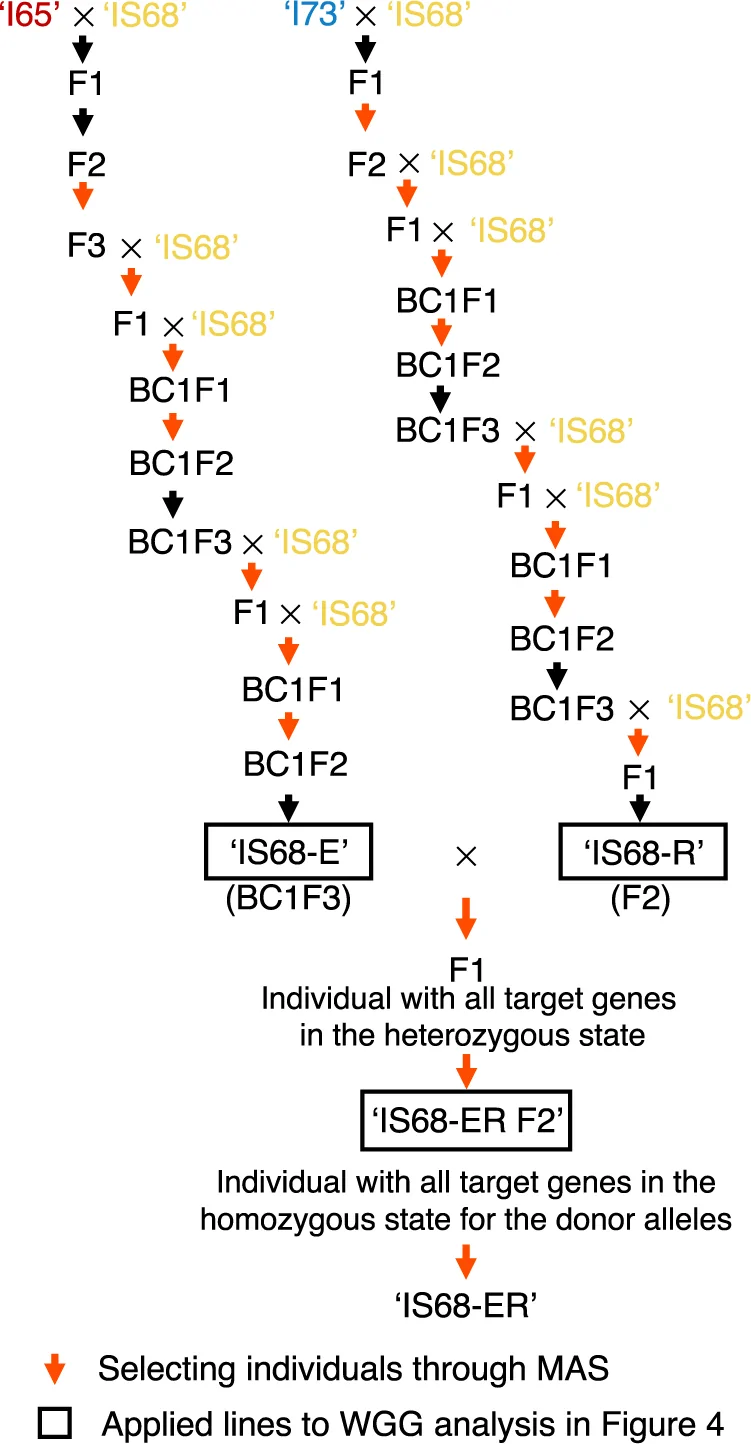
Schematic diagram of the breeding process for ‘IS68-ER’. Arrows indicate the progression of each generation during crossing and selection.

**Fig. 4.**
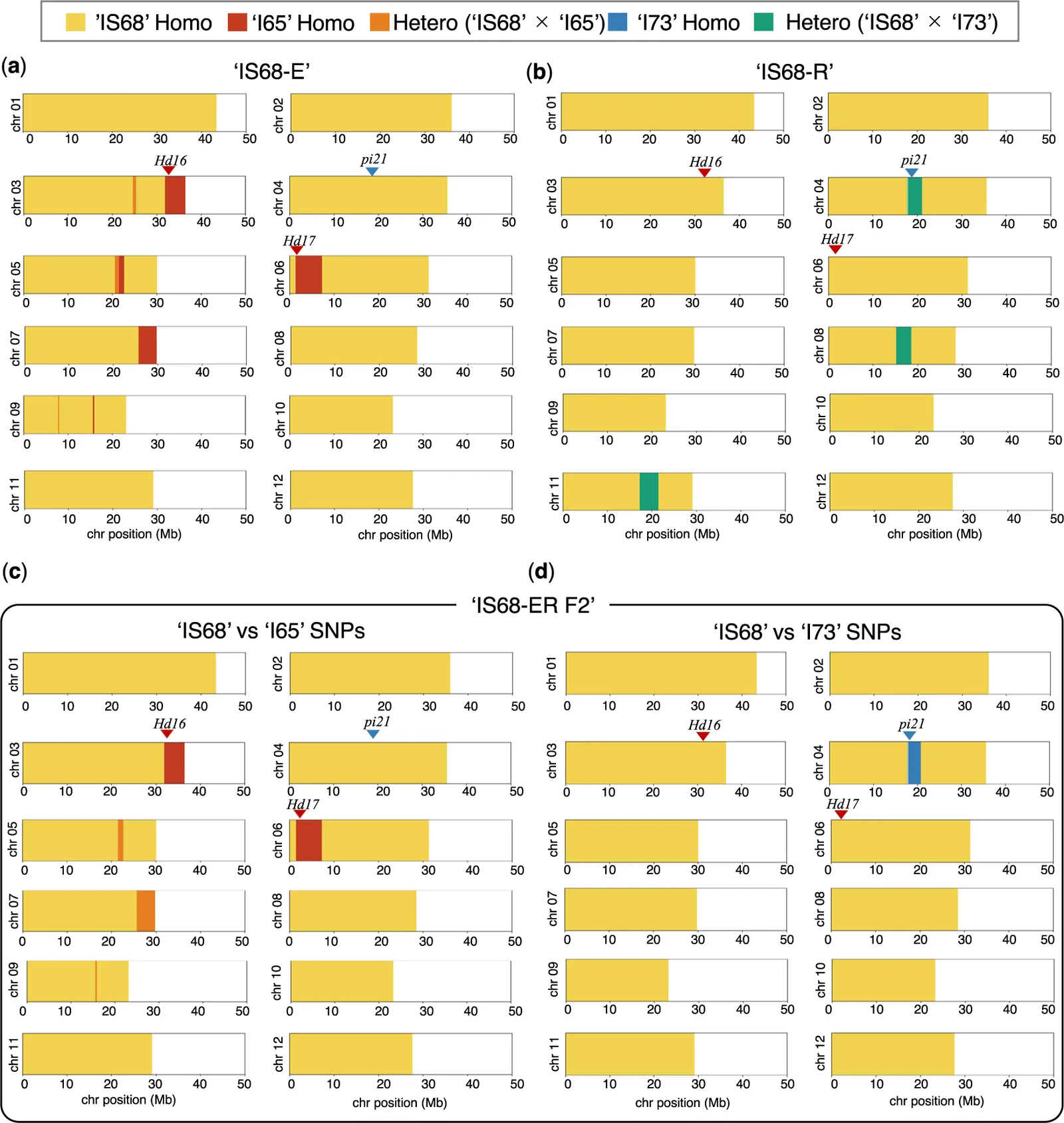
Application of WGG to ‘IS68-E’, ‘IS68-R’, and ‘IS68-ER’. WGG analysis visualized the genotype composition in each genomic region across all 12 rice chromosomes. Different genotypes containing alleles derived from the donor parents, ‘I65’ and ‘I73’, are shown in different colors. Red and blue triangles on the chromosomal bars indicate the positions of the target genes derived from ‘I65’ and ‘I73’, respectively. Parameters used for analysis: minimum depth >6; for sliding-window analysis, window size = 2 Mb, step size = 100 kb, and ≥3 SNPs per window. (a) ‘IS68-E’. (b) ‘IS68-R’. (c, d) ‘IS68-ER’ analyzed using SNPs detected between ‘IS68’ and ‘I65’ (c) and between ‘IS68’ and ‘I73’ (d). In (c), all SNP positions detected between ‘IS68’ and ‘I73’ were manually substituted with the ‘IS68’ homozygous genotype, and in (d), all SNP positions detected between ‘IS68’ and ‘I65’ were manually substituted with the ‘IS68’ homozygous genotype.

**Fig. 5.**
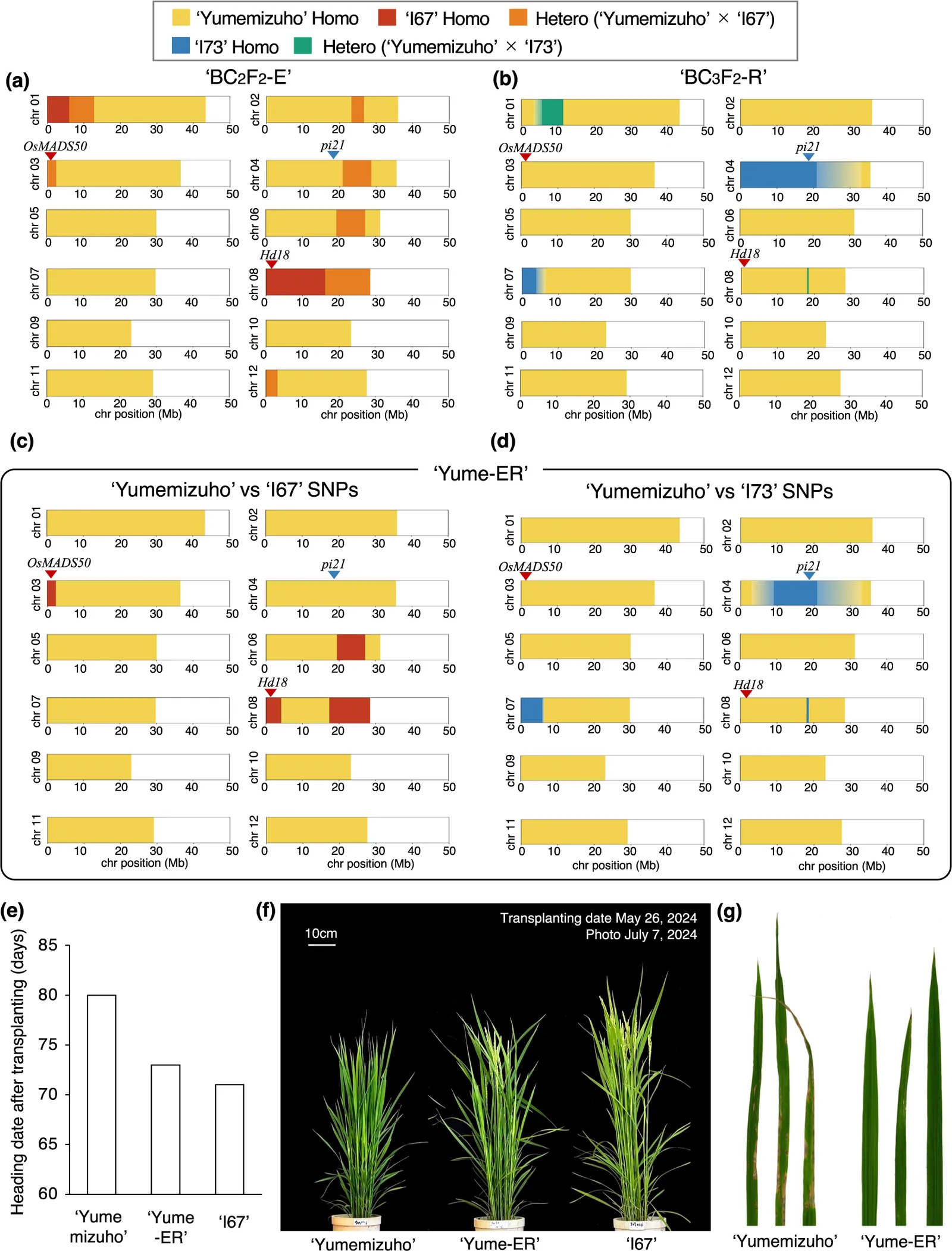
Rapid breeding of ‘Yume-ER’. (a–d) Application of WGG to ‘BC_2_F_2_-E’, ‘BC_3_F_2_-R’, and ‘Yume-ER’. In (c), all SNP positions detected between ‘Yumemizuho’ and ‘I73’ were manually substituted with the ‘Yumemizuho’ homozygous genotype, and in (d), all SNP positions detected between ‘Yumemizuho’ and ‘I67’ were manually substituted with the ‘Yumemizuho’ homozygous genotype. Parameters used for analysis: minimum depth >6; for sliding-window analysis, window size = 2 Mb, step size = 100 kb, and ≥3 SNPs per window. (e) Comparison of heading date among ‘Yumemizuho’, ‘Yume-ER’, and ‘I67’. (f) Photograph taken on July 7, 2024, at the heading stage of ‘Yume-ER’. (g) Leaves one month after artificial blast inoculation.

**Fig. 6.**
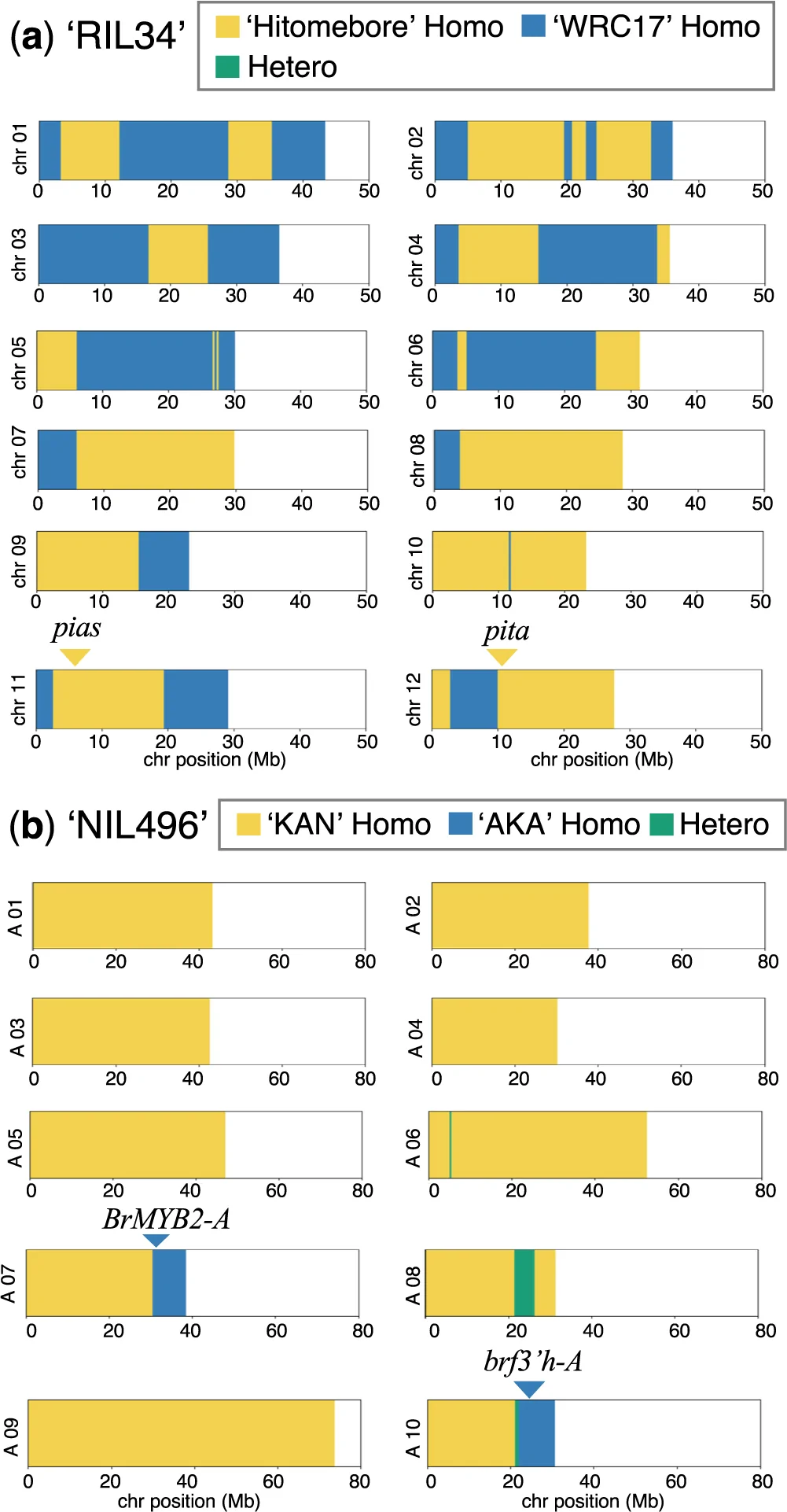
Application of WGG to RIL and heterozygous plants. (a) Application of WGG to an RIL developed from a cross between rice cultivars ‘Hitomebore’ and ‘WRC17’. The yellow triangles indicate positions expected to show the ‘Hitomebore’-homozygous genotype in the published study (Shimizu *et al.* 2022). (b) Application of WGG to ‘NIL496’. ‘NIL496’ was developed from a cross between ‘AKA’, used as the donor parent carrying *BrMYB2-A* and *brf3’h-A* (blue triangles), and ‘KAN’ as the recurrent parent. Parameters used for both analyses: minimum depth >6; for sliding-window analysis, window size = 2 Mb, step size = 100 kb, and ≥3 SNPs per window.

**Fig. 7.**
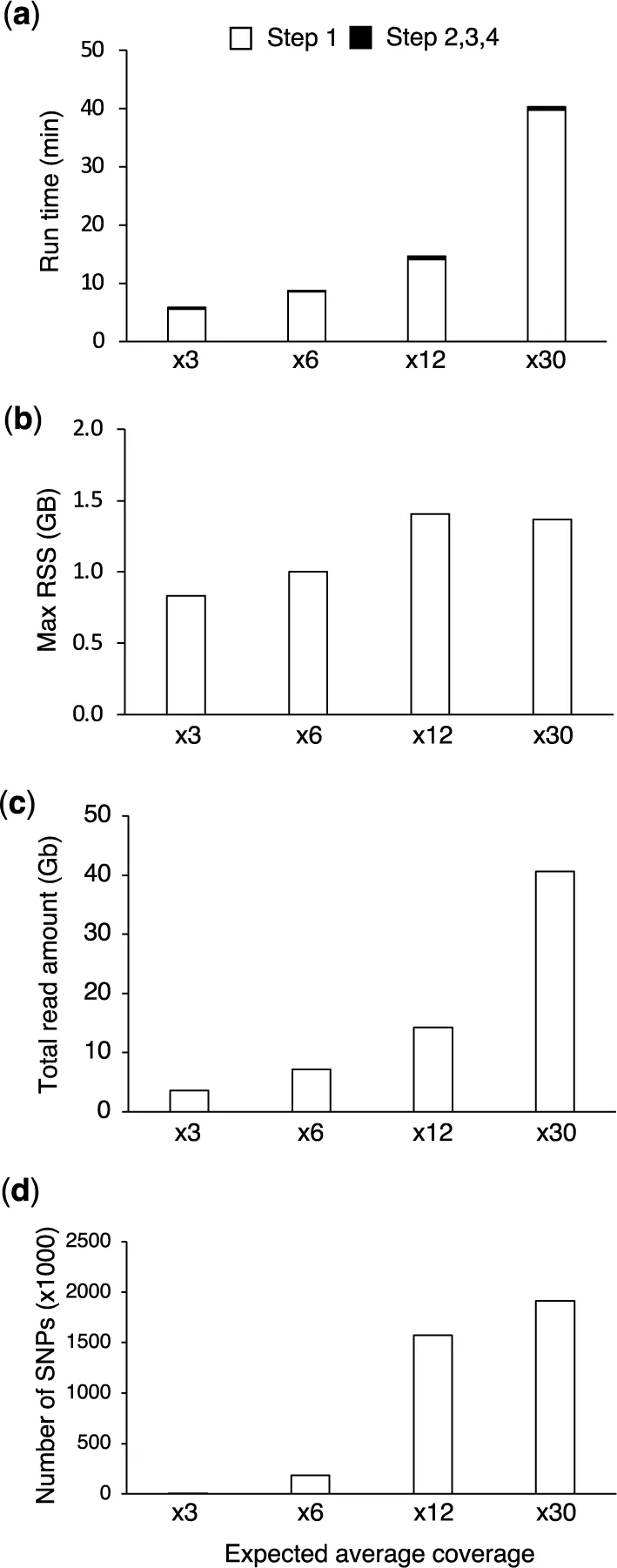
Performance validation of WGG. Comparison of processing time and memory usage across different sequencing coverage depths in the RIL sample used in Fig. 6. The average coverage depth of the RIL sample using the original read data was 30×. For comparison, the original reads were subsampled to adjust the coverage to 3×, 6×, 12×, and 30×. For the 3×, 6×, and 12× datasets, the read numbers of the parental lines ‘Hitomebore’ and ‘WRC17’ were also reduced to achieve the same coverage depths. For the 30× dataset, using the original read data, the average coverage depths of ‘Hitomebore’ and ‘WRC17’ were 60× and 28×, respectively. (a) Runtime at each step of the analysis pipeline. (b) Maximum memory usage (Max RSS). Values represent means obtained from three independent replicates. (c) Total read amount (Gb) of input sequencing data including both the RIL sample and the parents, ‘Hitomebore’ and ‘WRC17’. (d) Number of SNPs detected between ‘Hitomebore’ and ‘WRC17’.
