# Supplementary material for: WGG: a graphical tool for visualizing genome structures using SNPs detected by next-generation sequencing
Source: Breed Sci. 2026 Jun 11;76(3):217–28. doi: 10.1270/jsbbs.25080 (PMC13709638; doi:10.1270/jsbbs.25080)
Supplement: Supplementary file 1 [file 76_217_s1.pdf]

Takata et al./Breeding Science
doi: 10.1270/jsbbs.25080

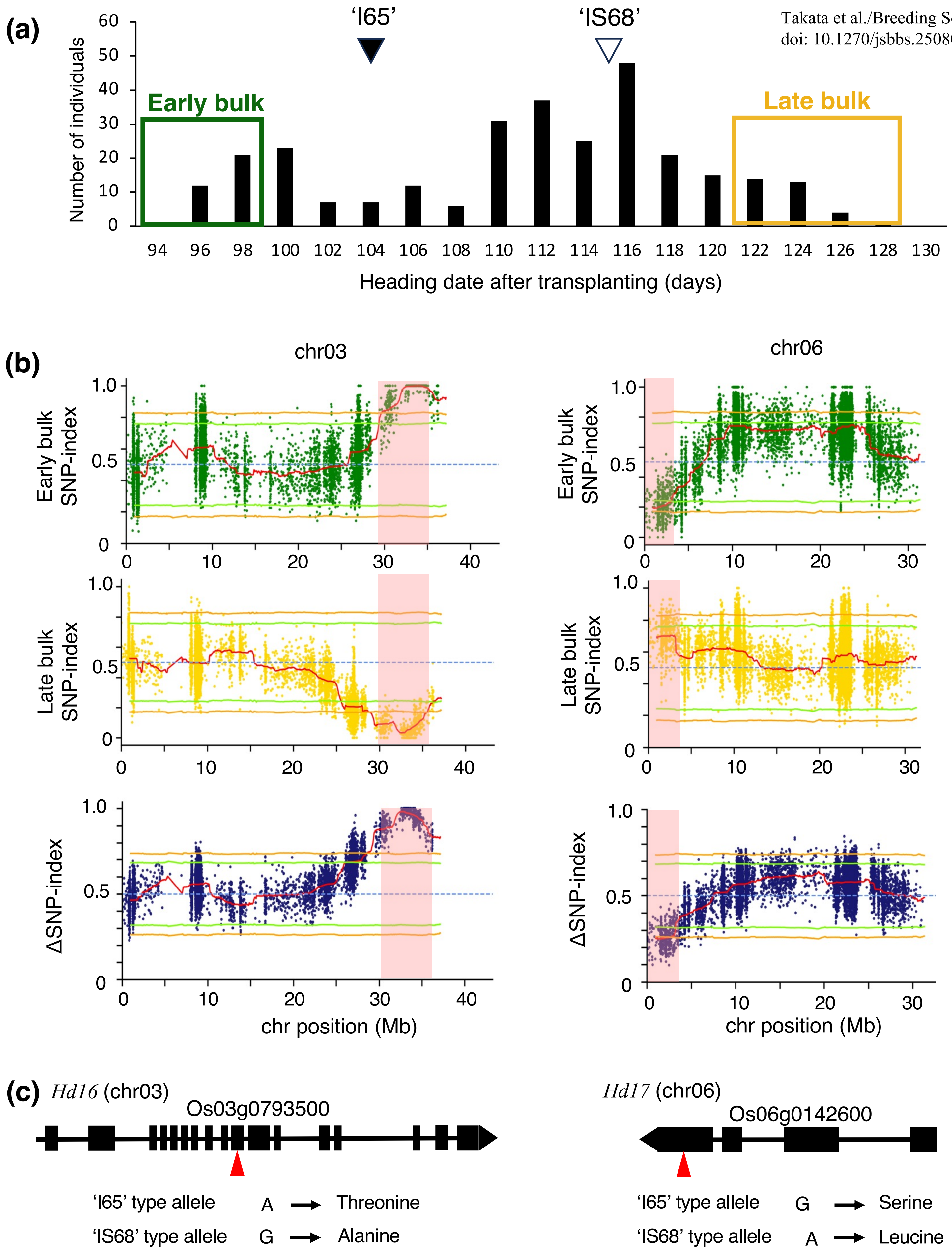

Supplemental Figure 1. Genetic analysis for identifying the genes responsible for the difference in heading date between 'IS68' and 'I65'.

(**a**) Frequency distribution of F_2 progeny derived from a cross between 'IS68' and 'I65'. The 20 individuals used for QTL-seq analysis as early- and late-heading bulks are indicated by green and yellow boxes, respectively. (**b**) QTL-seq plots for chromosomes 3 and 6. The red-highlighted regions indicate candidate genomic regions exceeding the 95% confidence interval of the SNP-index simulated from the hypothetically random bulk sample. (**c**) SNPs indicated by red triangles represent those responsible for the functional differences between the two alleles of *Hd16* and *Hd17*.

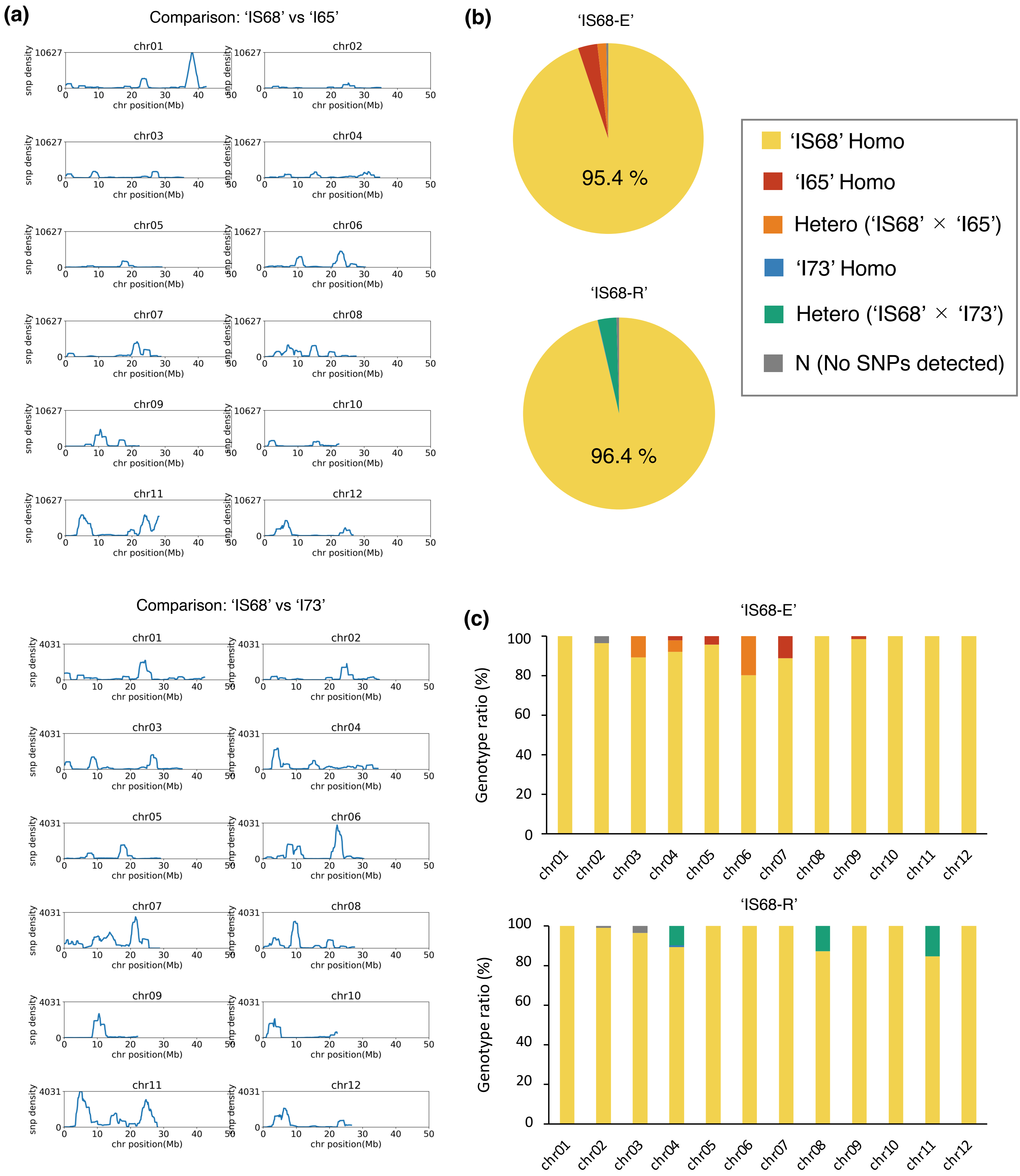

Supplemental Figure 2. Supplementary output data generated by WGG.
(**a**) Number of SNPs in each genomic window across the genome in the sliding-window analysis. (**b**) Total proportion of each genotype across the entire genome. (**c**) Proportion of each genotype on each chromosome.

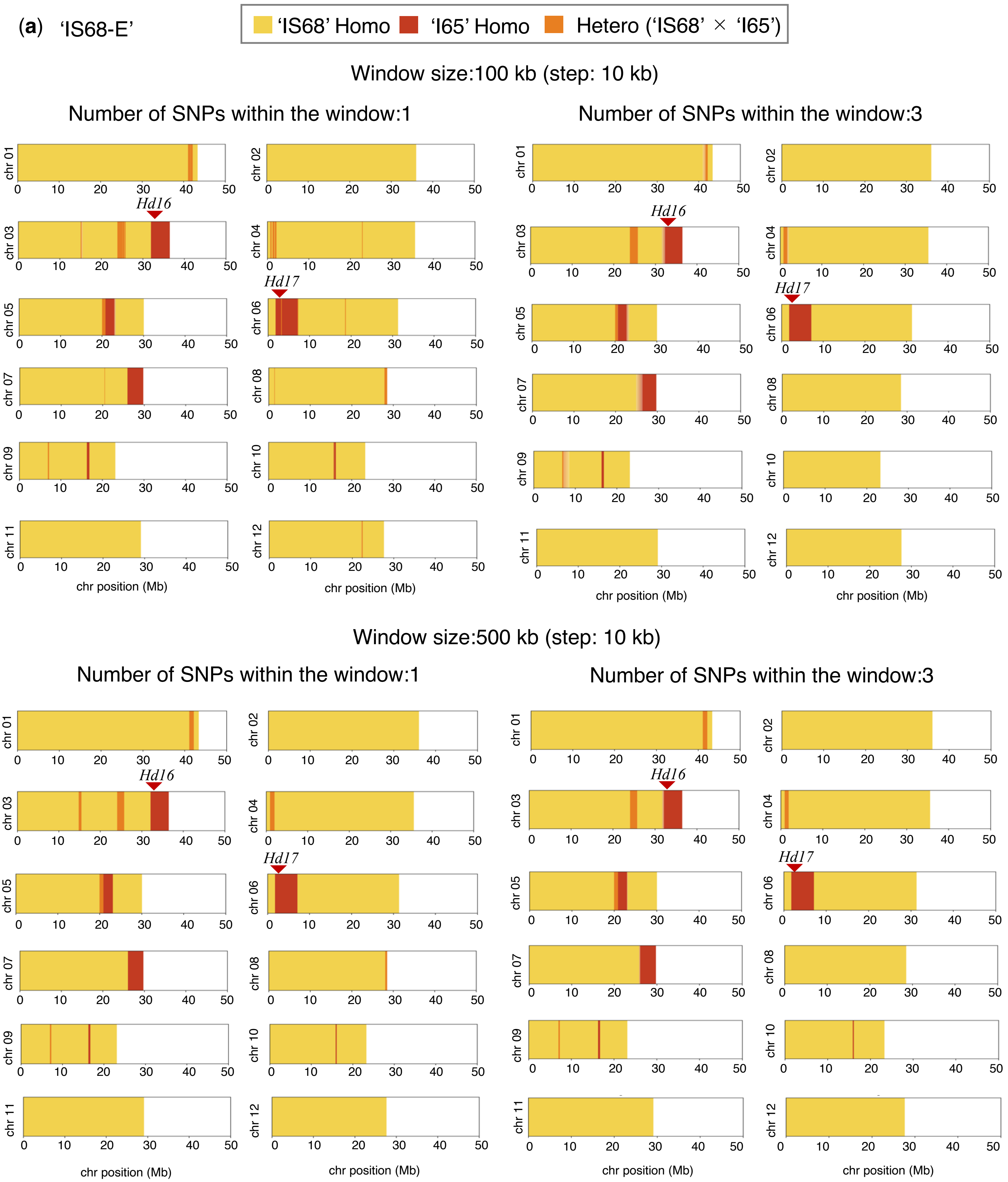

Supplemental Figure 3. (continue…)

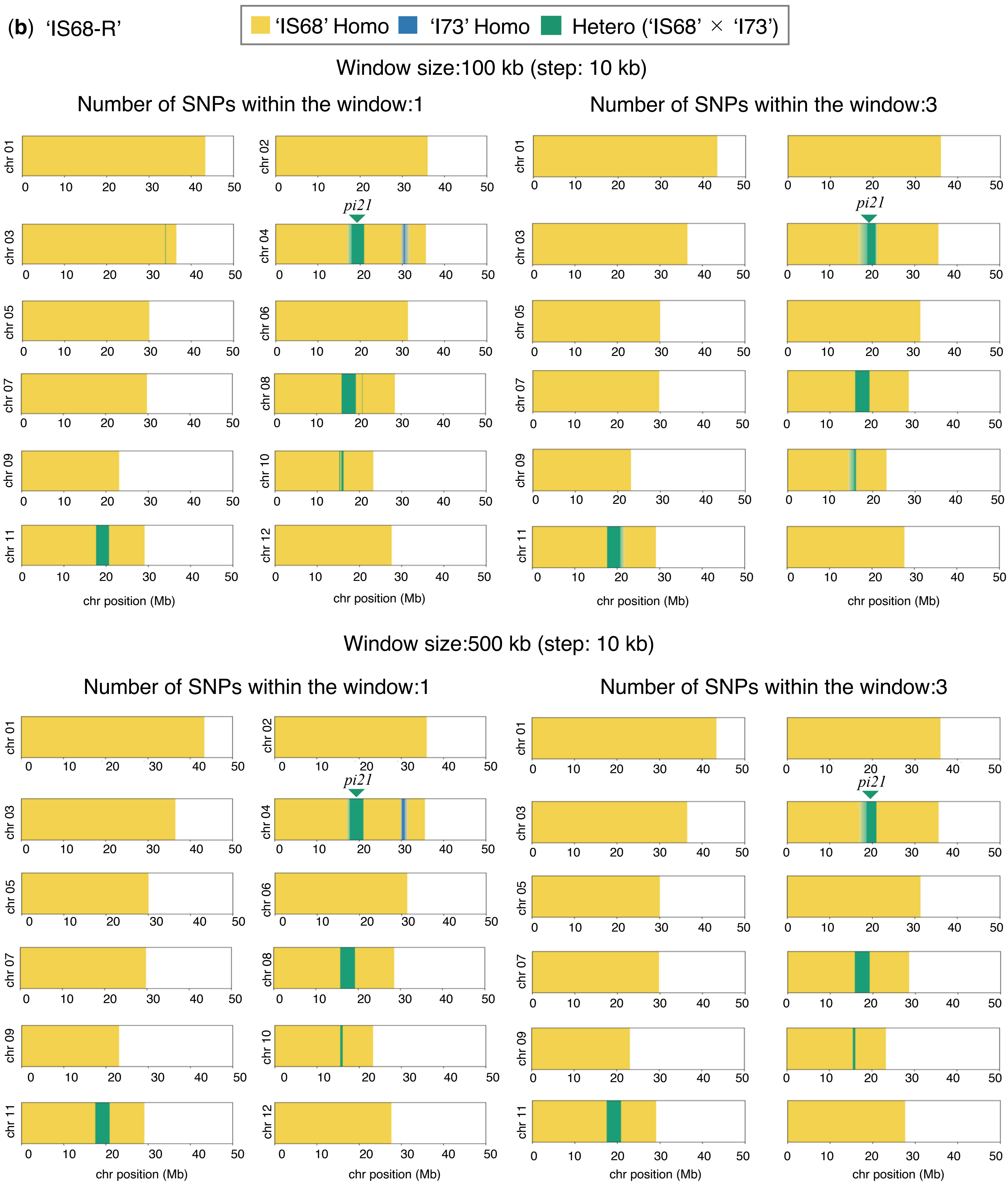

Supplemental Figure 3. (continue…)

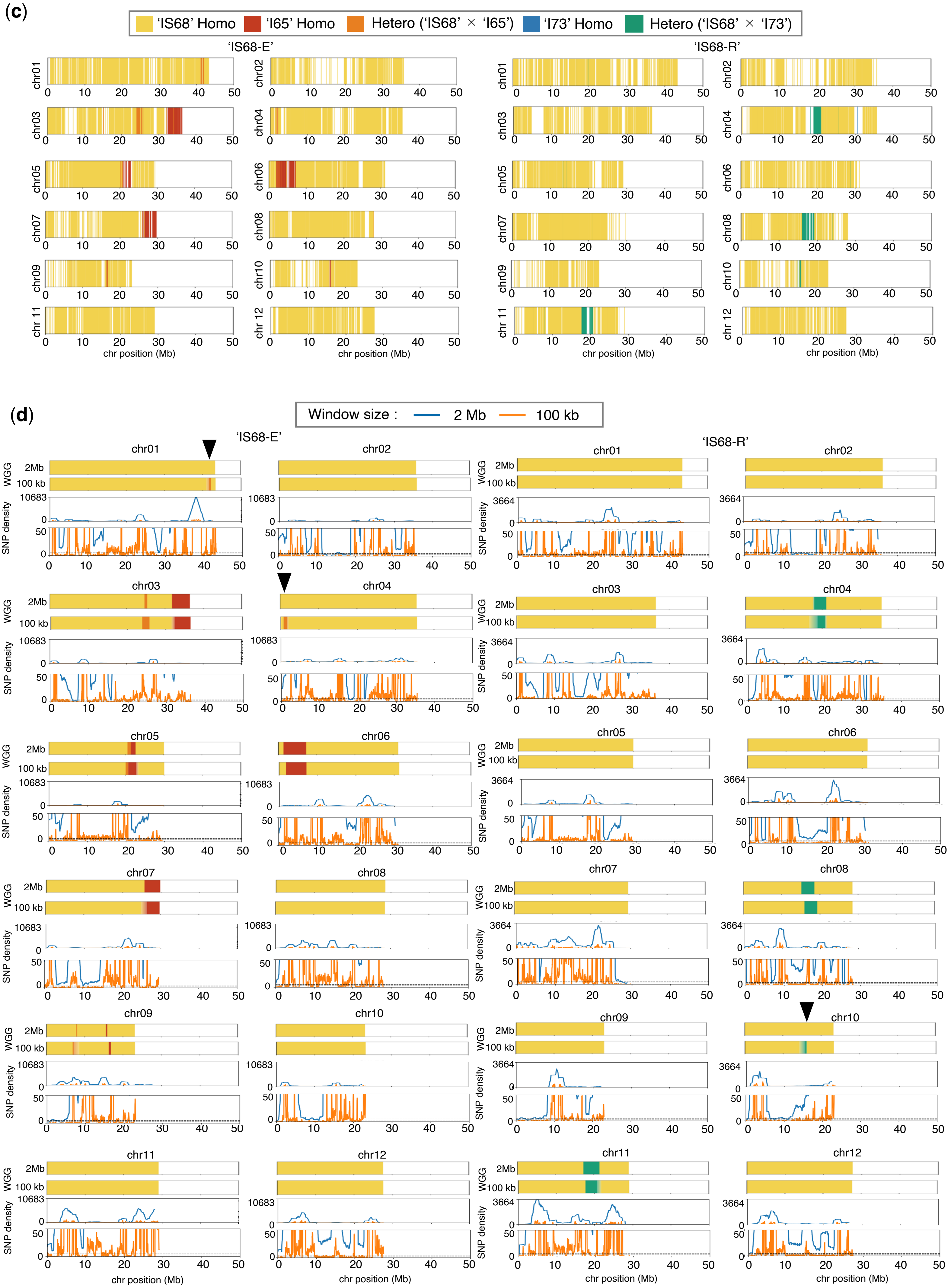

Supplemental Figure 3. (continue…)

Supplemental Figure 3. Effect of sliding-window parameters on WGG analysis.

Genome structures inferred by WGG using different sliding-window sizes and minimum numbers of SNPs per window in 'IS68-E' (**a**) and 'IS68-R' (**b**). (**c**) Visualization of genotypes at all SNP positions in 'IS68-E' and 'IS68-R', shown as colored lines. (**d**) Line plots showing SNP density in each genomic region between the parental cultivars, calculated using window sizes of 100 kb and 2 Mb. Black arrowheads indicate positions where irregular genotypes were detected when a window size of 100 kb was applied.

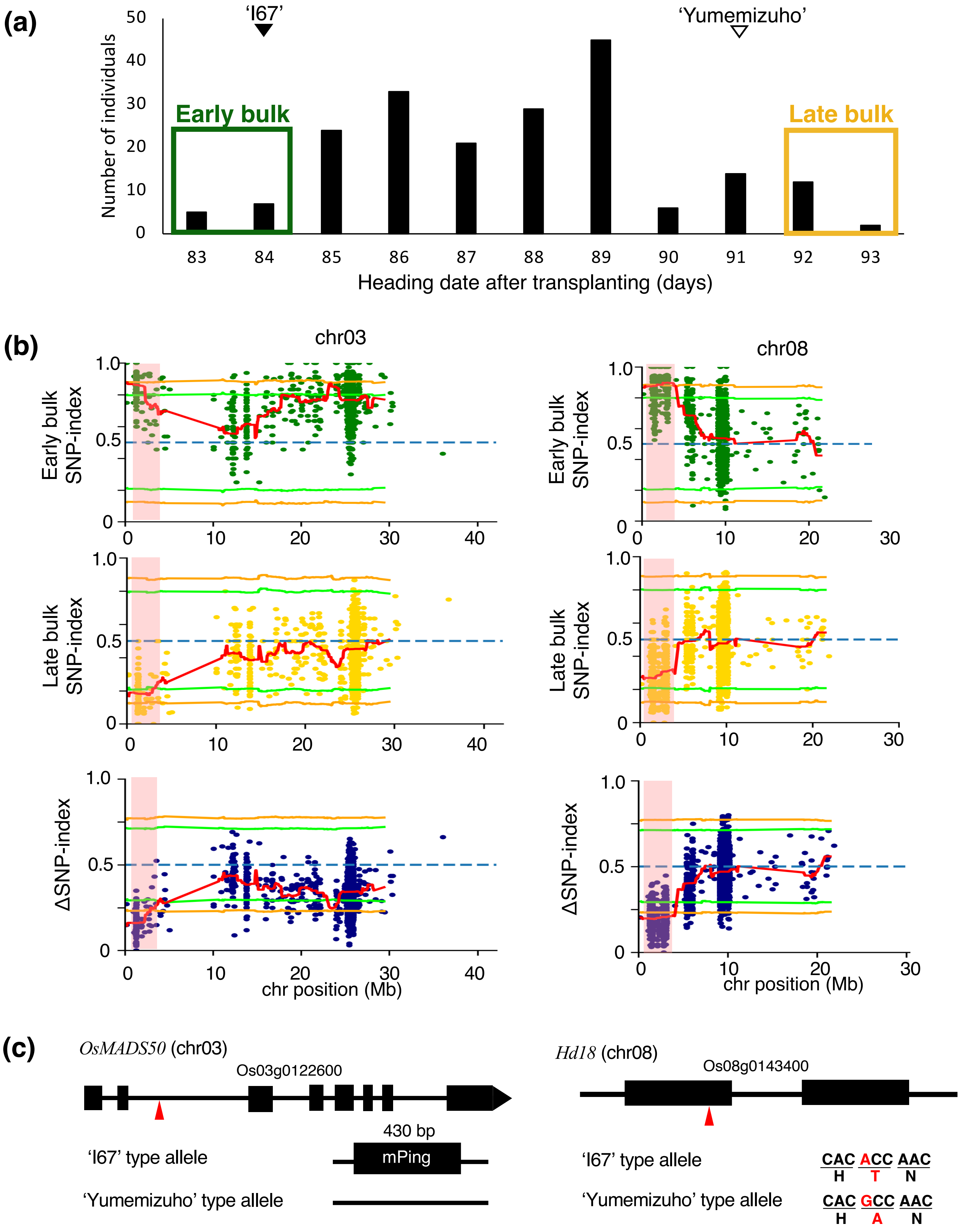

Supplemental Figure 4. Genetic analysis for identifying the genes responsible for the difference in heading date between 'Yumemizuho' and 'I67'.
(**a**) Frequency distribution of F_3 progeny derived from a cross between 'Yumemizuho' and 'I67'. The 15 individuals used for QTL-seq analysis as early- and late-heading bulks are indicated by green and yellow boxes, respectively. (**b**) QTL-seq plots for chromosomes 3 and 8. The red-highlighted regions indicate candidate genomic regions exceeding the 95% confidence interval of the SNP-index simulated from hypothetically randomized bulk samples. (**c**) Red triangles indicate the positions responsible for the functional differences between the two alleles of *OsMADS50* and *Hd18*.

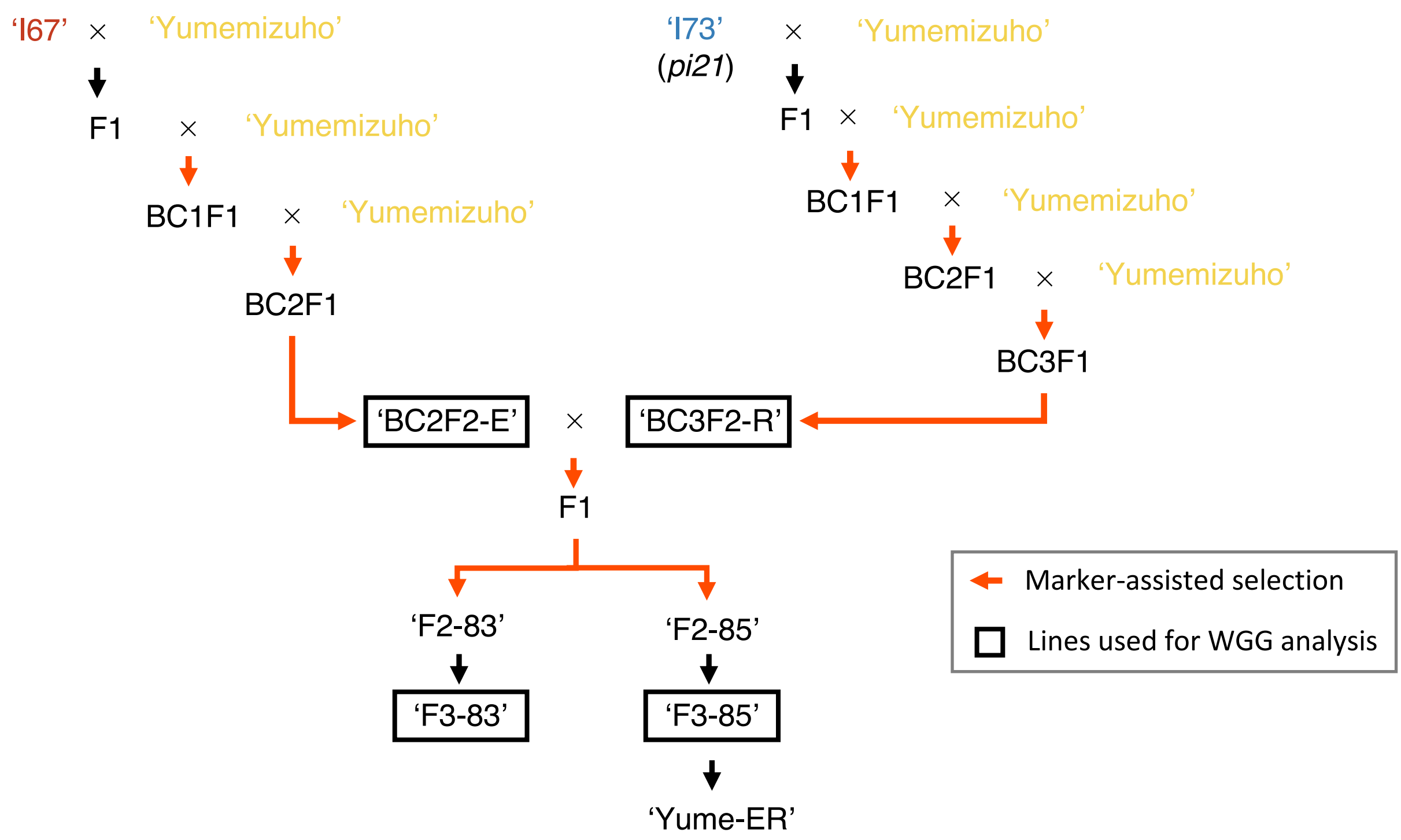

Supplemental Figure 5. Schematic diagram of the breeding process for developing ‘Yume-ER’.

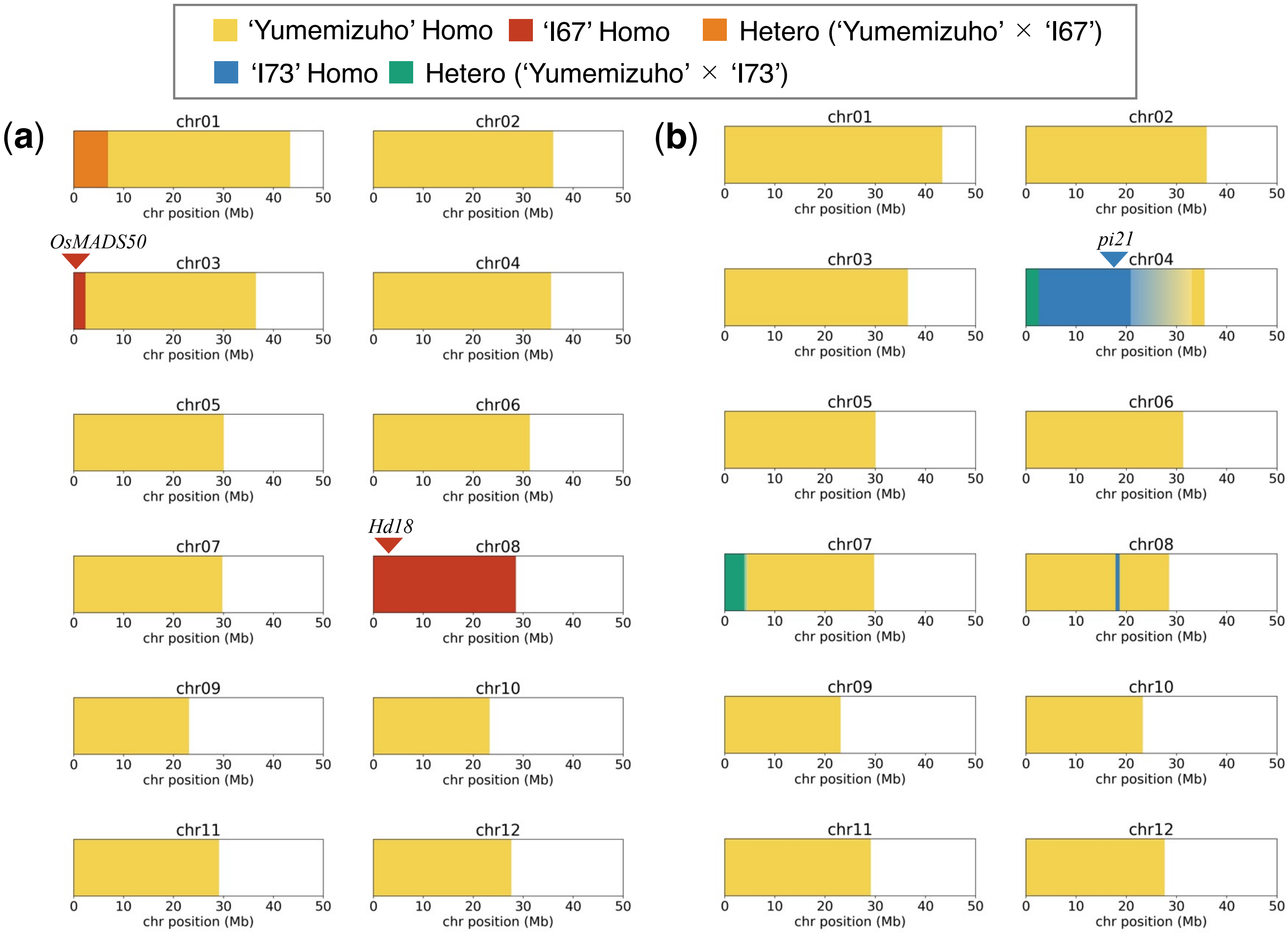

Supplemental Figure. 6 WGG result of 'F3-83'.
'F3-83' was analyzed using SNPs detected between 'Yumemizuho' and 'I67' (**a**) and between 'IS68' and 'I73' (**b**). In (a), all SNP positions detected between 'Yumemizuho' and 'I73' were manually substituted with the 'Yumemizuho' homozygous genotype, and in (b), all SNP positions detected between 'Yumemizuho' and 'I67' were manually substituted with the 'Yumemizuho' homozygous genotype. Parameters used for analysis: minimum depth > 6; for sliding-window analysis, window size = 2 Mb, step size = 100 kb, and ≥ 3 SNPs per window. Red and blue triangles on the chromosomal bars indicate the positions of the target genes derived from 'I67' and 'I73', respectively.

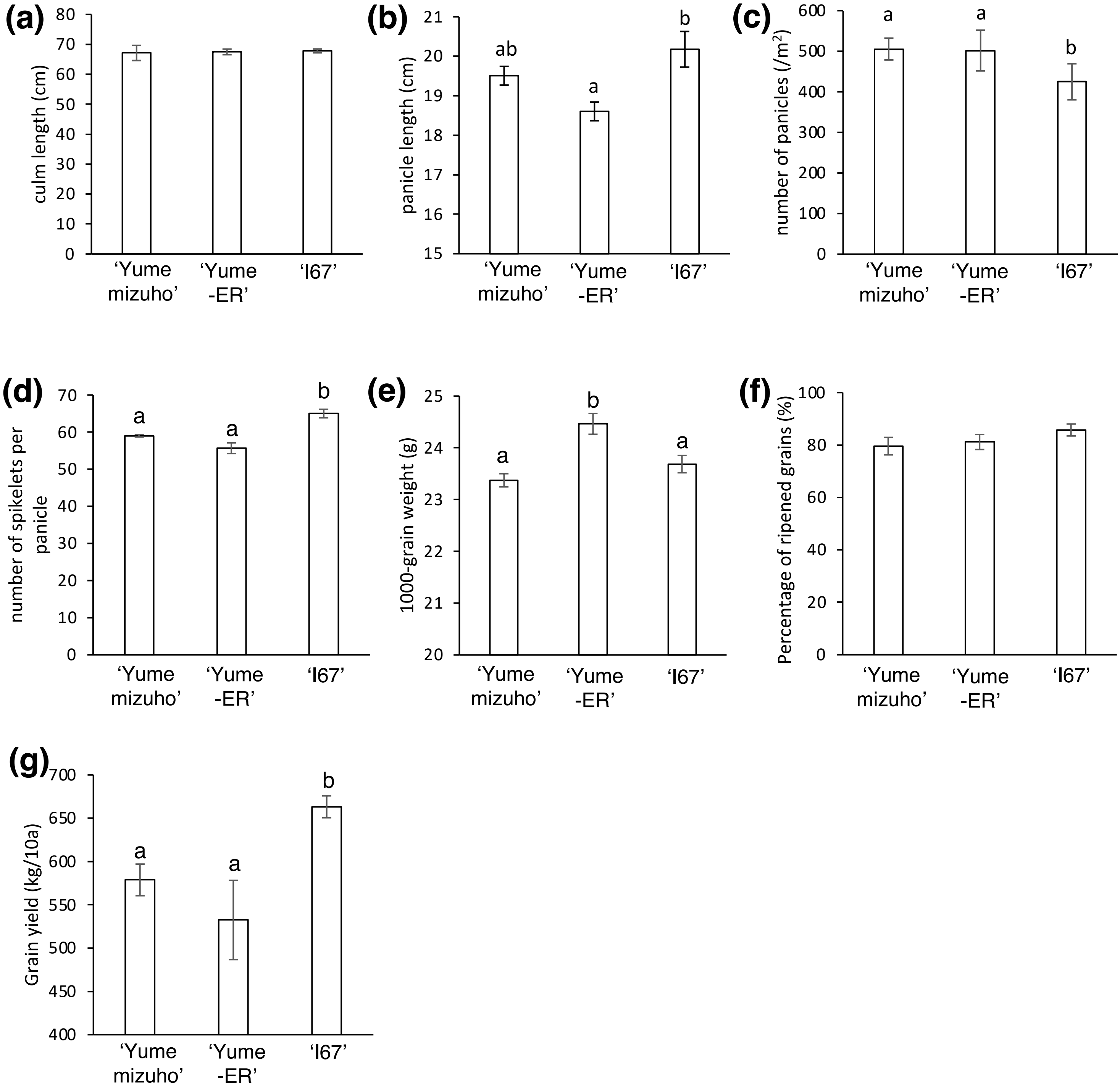

Supplemental Figure 7. Evaluation of agronomic traits in 'Yume-ER'.
Agronomic traits were compared among ten individuals of 'Yume-ER', 'Yumemizuho', and 'I67'. All plants were cultivated in the experimental field of Ishikawa Prefectural University in 2025. Basal fertilizer was applied at a rate of 8 kg N per 10 a. (**a**) Culm length, (**b**) panicle length, (**c**) number of panicles per m^2, (**d**) number of spikelets per panicle, (**e**) 1,000-grain weight, (**f**) percentage of ripened grains, and (**g**) grain yield. Bars represent means ± SE (n = 3). Different letters indicate significant differences among lines according to Tukey's HSD test (p < 0.05).

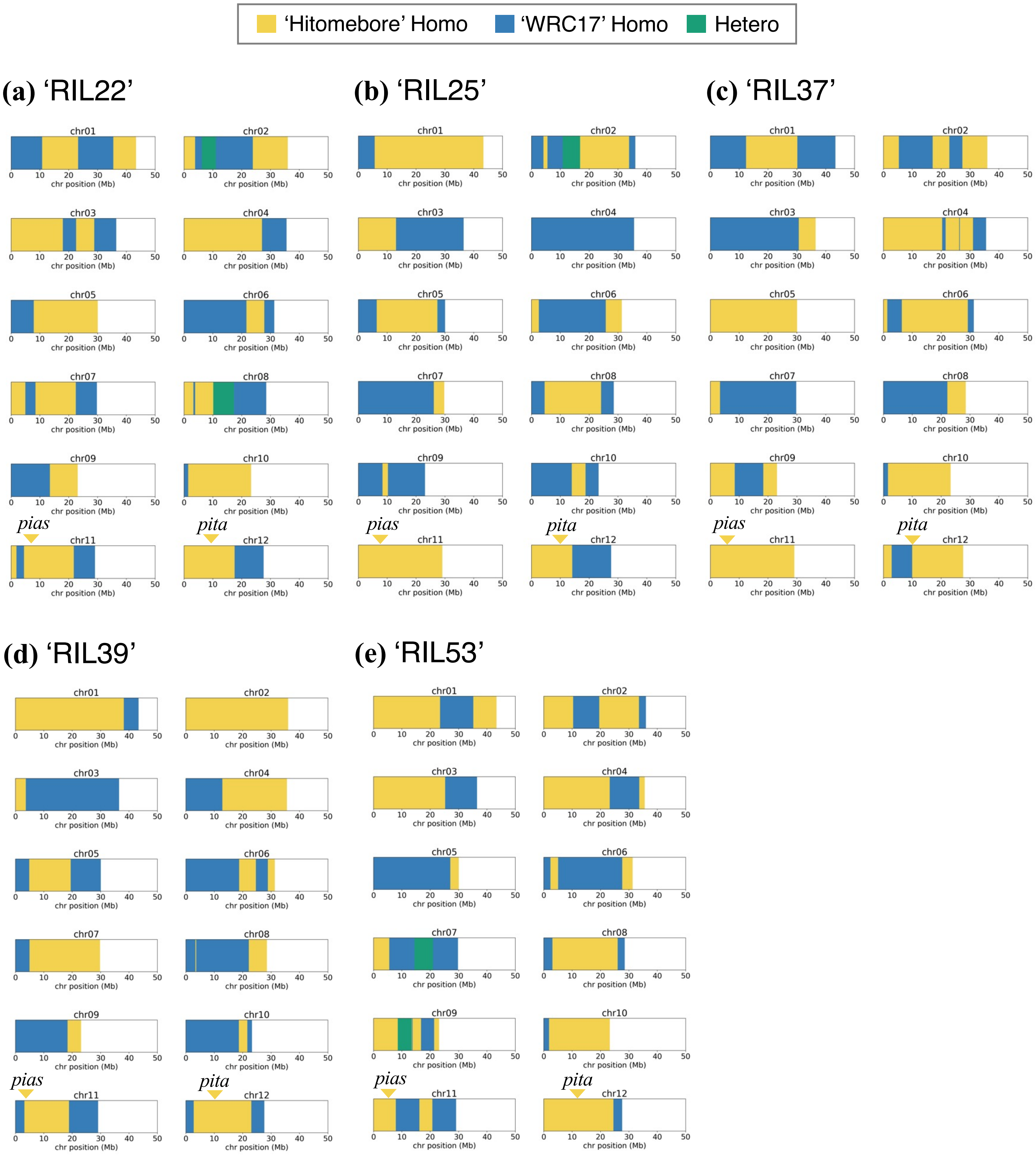

Supplemental Figure 8. Application of WGG to RILs developed from a cross between rice cultivars 'Hitomebore' and 'WRC17'.

Yellow triangles indicate the positions expected to show the 'Hitomebore'-homozygous genotype, as reported in the published study (Shimizu *et al*., 2022). Parameters used were the same as those in Figure 6.

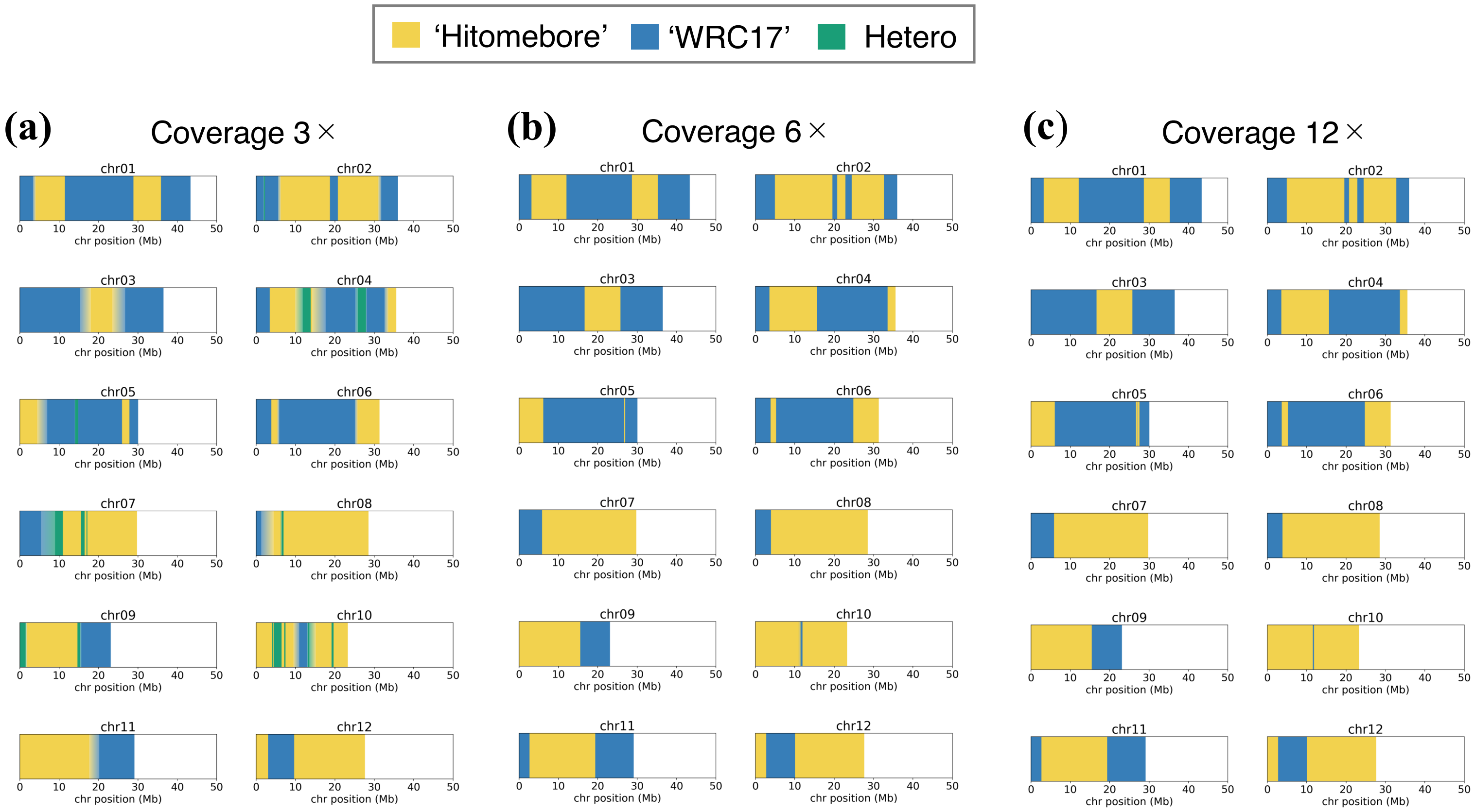

Supplemental Figure 9. Effect of coverage depth on WGG analysis.
WGG was applied to the RILs described in Fig. 6a using different amounts of short-read data.

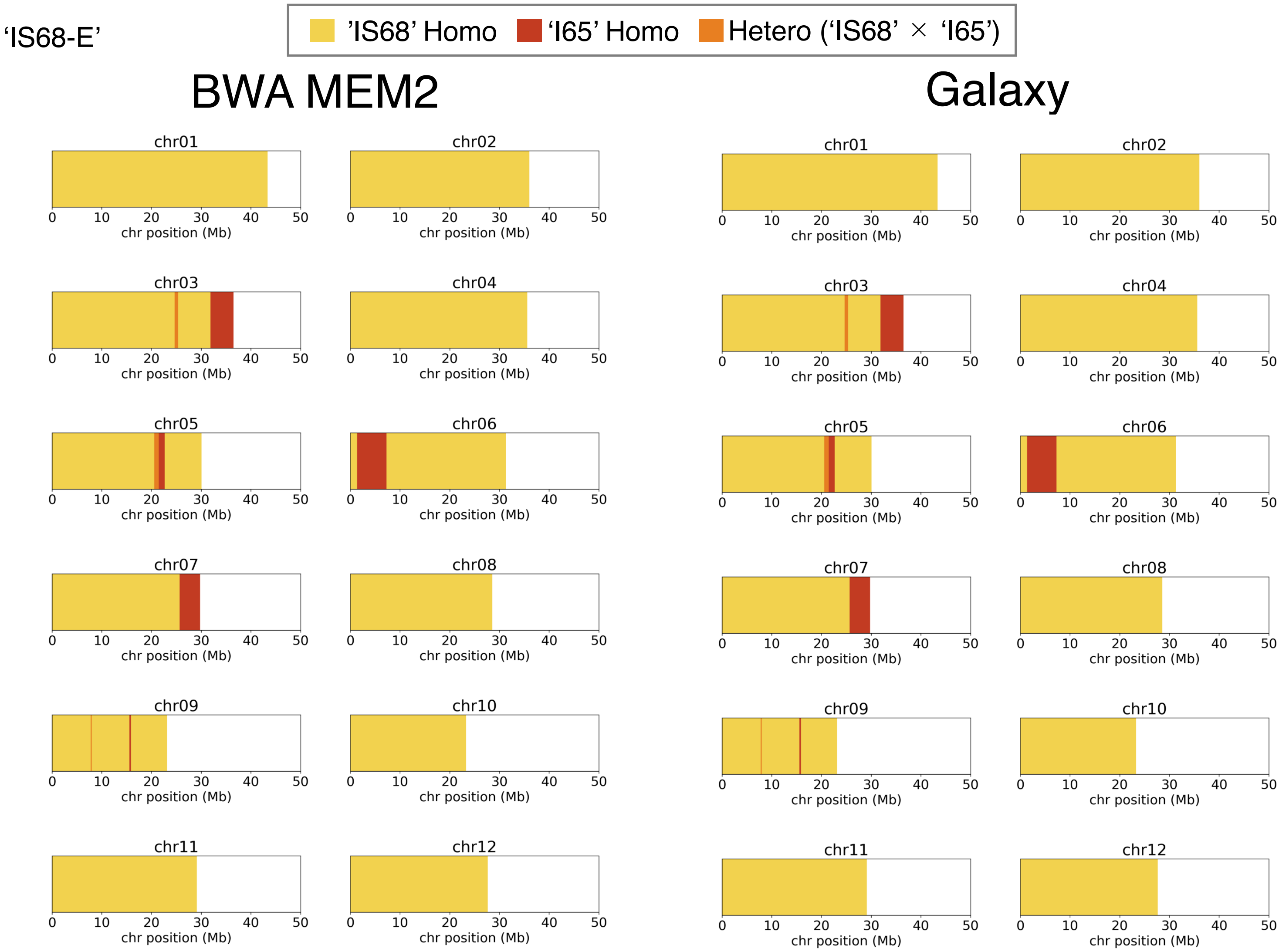

Supplemental Figure 10. WGG results using BAM files generated by bwa-mem2 on the command line or through the Galaxy platform.
In Galaxy, the ‘BWA-MEM2’ tool was used with default settings.
