# Supplementary material for: WGG: a graphical tool for visualizing genome structures using SNPs detected by next-generation sequencing
Source: Breed Sci. 2026 Jun 11;76(3):217–28. doi: 10.1270/jsbbs.25080 (PMC13709638; doi:10.1270/jsbbs.25080)
Supplement: Supplementary file 2 [file 76_217_s2.pdf]

doi: 10.1270/jsbbs.25080

Supplemental Table 1. Summary of Illumina paired-end reads sequenced for this study.

Sample name	Total sequences (Gb)	Read length(bp)	Accession number	Reference
'IS68'	16.8	150	DRR792840	obtained in this study
'I65'	12.7		DRR792841	
'I73'	3.6		DRR792842	
'I65' vs. 'IS68' Ebulk	10.3		DRR792843	
'I65' vs. 'IS68' Lbulk	16.9		DRR792844	
'IS68-E'	5.2		DRR792845	
'IS68-R'	4.7		DRR792846	
'IS68-ER'	5.4		DRR792847	
'Yumemizuho'	7.4		DRR792848	
'I67'	4.7		DRR792849	
'Yumemizuho' vs. 'I67' E-bulk	8.7		DRR792850	
'Yumemizuho' vs. 'I67' L-bulk	7.0		DRR792851	
'BC2F2-E'	6.2		DRR792852	
'BC3F2-R'	11.6		DRR792853	
'Yume-ER'('F3-85')	6.8		DRR792854	
'F3-83'	8.3		DRR792855	
'NIL496'	7.6		DRR792856	
'AKA'	9.5	150	DRR218927	Segawa *et al.* 2021
'KAN'	18.5		DRR218928	
'RIL22'	11.5	150	DRR319691	Shimizu *et al.* 2022
'RIL25'	5.4		DRR319692	
'RIL34'	6.2		DRR319693	
'RIL37'	12.8		DRR319694	
'RIL39'	10.0		DRR319695	
'RIL53'	11.5		DRR319696	
'Hitomebore'	23.3	100	DRR319689	
'WRC17'	11.1	150	DRR319690	

Supplemental Table 2. Summary of the primers used in this study.

Name	Primer sequence (5'–3')	Restriction enzyme	Description
Fw1	TACGTCTAAGCAGCCCGGA		*OsMADS50* indel marker
Rv1	GCAAGTTTAATAGCAGAGCTCAC		
Fw2	TACCTGTAATTGGGCCCAATCACCG	EcoT14I	*Hd18* CAPS marker
Rv2	GTCACATCGGCCGTTTGACC		
Fw3	GGTAGCGTCTACCACACTCTGCATC	XbaI	*Hd18* CAPS marker
Rv3	ACACCTCACTACCATGCAGCA		
Fw4	TTGTGTTTTGGAGACTGCGCC		*Hd16* indel marker
Rv4	AGCTTATGTGGAGGAGGGAGAT		
Fw5	TGTTCGACTACAGGTGCCGG		*Hd17* tetra ARMS marker
Rv5	AGAATGCCGGCATGTTTTATTTTCC		
IS68_Rv5	GGCTTATAGACAAGGCCTTGCA		
I65_Rv5	TGTCCCTGTCATGTCTCCCTC		
Fw6	CTTTGGGCAGCACCACTG		*pi21* indel marker designated by NARO
Rv6	GTACGACGTGAAGAACAACAGG		*pi21* InDel marker designed by ourselves
Fw7	TGACTGCATTGGAACTTGTGG		F_1-selection marker (‘IS68-E’ × ‘IS68-R’, Sanger sequencing) on chr11
Rv7	GTGGGTGGGCTAAAGATGGA		
Fw8	CGGCTACAAACAAGAGCAGAAG		F_1-selection marker (‘IS68-E’ × ‘IS68-R’, Sanger sequencing) on chr08
Rv8	ATGGGATGGGCAAATGGA		
Fw9	CTTTCGCCGTGCAAGTGGAC	EcoRV	Background-selection marker (CAPS marker) on 'IS68-ER' chr08
Rv9	TGACACGTAGAGGCTGTTCAGA		
Fw10	TGGCCGTTTCAGTTCCAACA	XbaI	Background-selection marker (CAPS marker) on 'IS68-ER' chr04
Rv10	GCCTCTCAGTGGAGCTGTGT		
Fw11	GCAACAACACTGCACAACCCT		Background-selection marker (Sanger sequencing) on 'IS68-ER' chr05
Rv11	GCGCGCTCTCTATGGGTTCC		
